# Synthesis of mixed musks *via* Eschenmoser–Tanabe fragmentation, enyne metathesis and Diels–Alder reaction as key steps†

**DOI:** 10.1039/d2ra01458k

**Published:** 2022-05-11

**Authors:** Sambasivarao Kotha, Arpit Agrawal, Yellaiah Tangella

**Affiliations:** Department of Chemistry, Indian Institute of Technology Bombay, Powai Mumbai 400076 Maharashtra India srk@chem.iitb.ac.in

## Abstract

Musk analogues containing different macrocyclic ring systems as well as different annulated ring systems were synthesised by a simple and useful strategy. This strategy includes Eschenmoser–Tanabe fragmentation, enyne metathesis and Diels–Alder reaction as key steps. Starting from easily available (*n*) macrocyclic ketones, (*n* + 3) macrocyclic systems were assembled using the basic organic reactions.

## Abstract 2

Musk analogues containing different macrocyclic ring systems as well as different annulated ring systems were synthesised by a simple and useful strategy.

## Graphical abstract

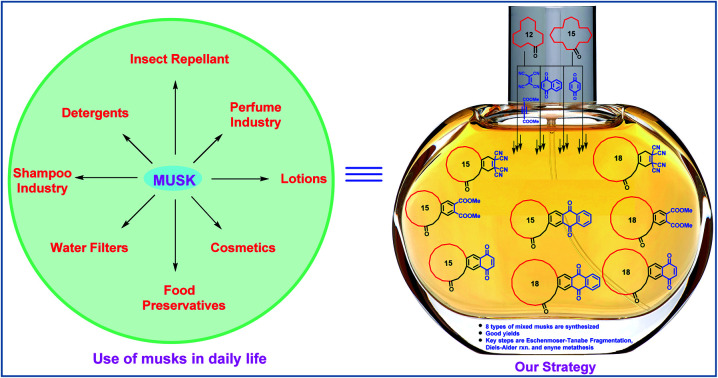

## Introduction

A musk is a compound with unusual fragrance properties.^1^ The market of the fragrance industry has gained momentum from the 20th century, and large quantities of synthetic musks have been synthesized in the world. In the flavour and fragrance industry, musks are used as perfume additives on a large scale.^2^ These compounds are also used as personal care products which include insect repellent, UV filters, preservatives and antimicrobial agents. There are a wide range of personal care products (PCPs) that contain synthetic musks, including lotions, shampoos, perfumes, softeners, air fresheners, washing powders *etc.*^3^

Among various synthetic fragrances available on the market, polycyclic musks (61%) and nitro musks (35%) play a dominant role in production volume.^4^ Most nitro musks have been withdrawn from the market due to their potential toxicity. Exposure to nitro musks is associated with an increased risk of tumorigenesis in mice. Evidence suggests that nitro musk itself is not genotoxic, but may increase genotoxicity of other chemicals. However, animal models of exposure to nitro musk have proven to be problematic because certain results are species-specific. It is also found that nitro musk compounds were not easily degraded, which makes them very stable and ubiquitous in the environment.^5^ After a sharp decline in the use and production of nitro musks, polycyclic musk compounds (PMCs) have become the leading commercial synthetic musks that now dominates the global market. Mainly, PMCs sold as Galaxolid® (HHCB) and Tonalide® (AHTN) account for approximately 95% of all fragrances in the perfume industry and are the most frequently detected PMCs in environmental compartments and living tissues at an environmentally suspicious level. Concerns about potential impacts relate not only to the environment, but also to food safety and thus to public health. Nonetheless, studies have been conducted to report potential oestrogenic and anti-oestrogenic effects in several PMCs, due to this reason their use in cosmetics is uncertain.^6^ Macrocyclic musks occupies a small part (3–4%) of the market and is used almost exclusively for perfumes. In 2017, the flavor and fragrance market was valued at $24.8 billion. Macrocyclic musks supports all consumer categories in the fragrance industry and has good environmental performance, renewable options and other sustainability resulting in high demand. As a result, both scientific and industrial synthetic chemists are continuously developing new strategies for the preparation of macrocycles. Desirable macrocycles include structures with one carbonyl unit and 13–19 carbon atoms.

**Fig. 1:**
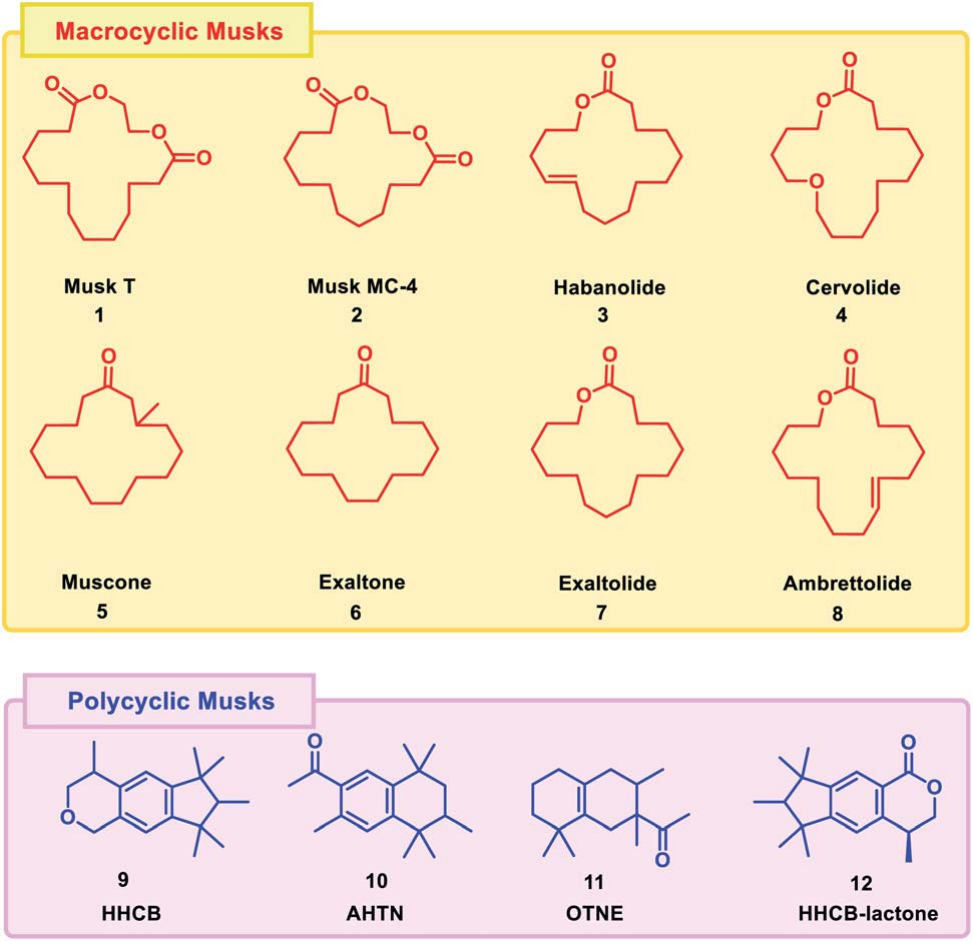
Representatives of macrocyclic and polycyclic musks.

These structural properties are known to produce the scent of musk, a well-known scent used in perfumes, colognes, and personal care products. Small structural changes to macrocycles, such as the incorporation of olefins, can create a characteristic odor profile. In addition, stereochemistry and the location of alkyl group substitutions or double bonds within the macrocycle skeleton can affect the resulting musk odor. Also due to their stronger odor, less amounts are needed to achieve the same level of performance as other synthetic musk varieties. Structural similarity of macrocyclic musks with that of the natural musks leads to the conclusion that these compounds appear to be more environment friendly and environmentally degradable than their predecessor class.^7^ Some of the examples of macrocyclic and polycyclic musks are given in Fig. 1.

**Scheme 1:**
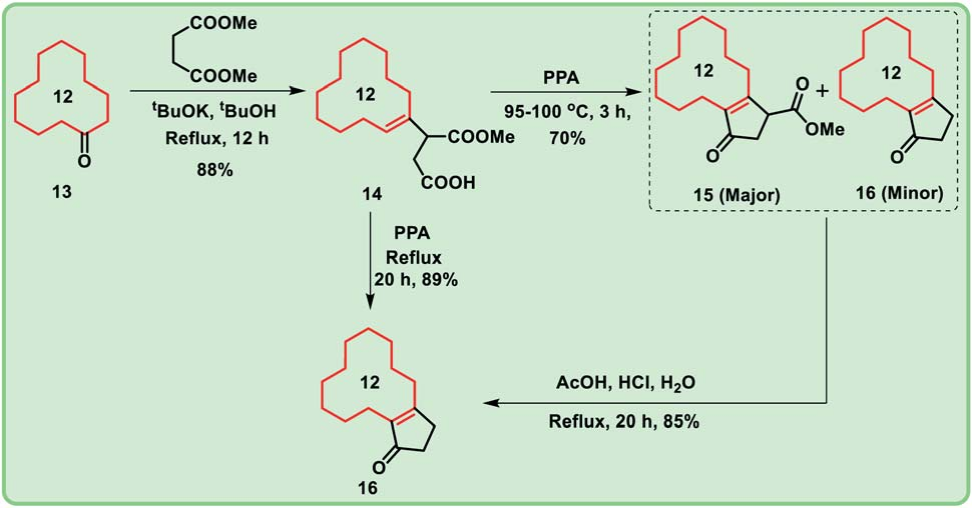
Formation of compound 16 from compound 14 exclusively.

**Scheme 2:**
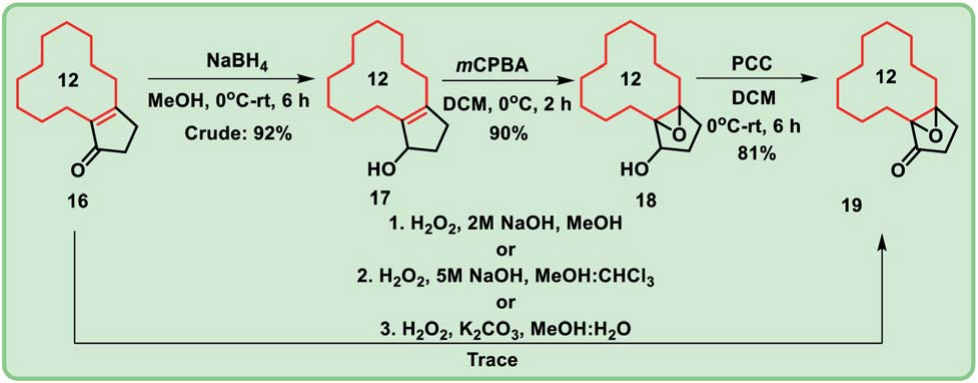
Synthesis of compound 19*via* 3 simpler known steps in order to increase the yield.

**Scheme 3:**
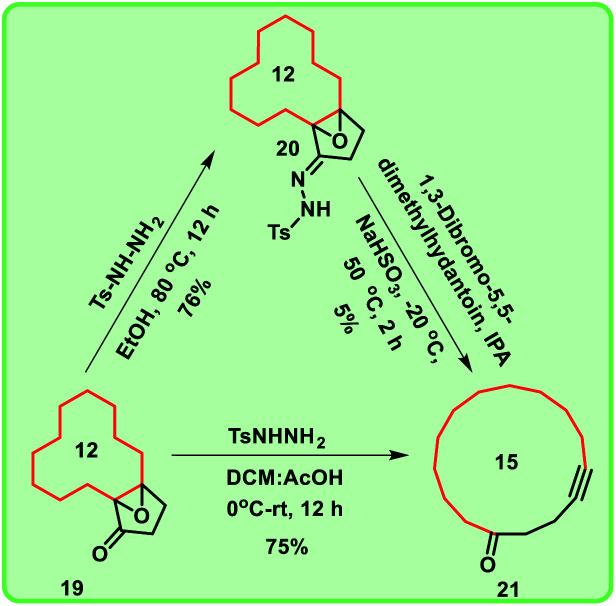
Synthesis of macrocyclic ring 15 using Eschenmoser–Tanabe fragmentation.

**Scheme 4:**
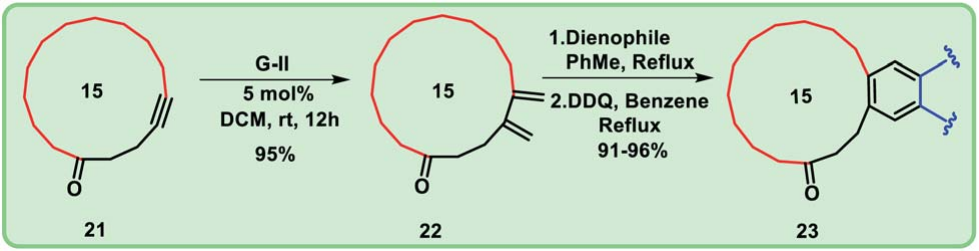
Synthesis of mixed musk using Diels–Alder cyclization followed by aromatization.

**Scheme 5:**
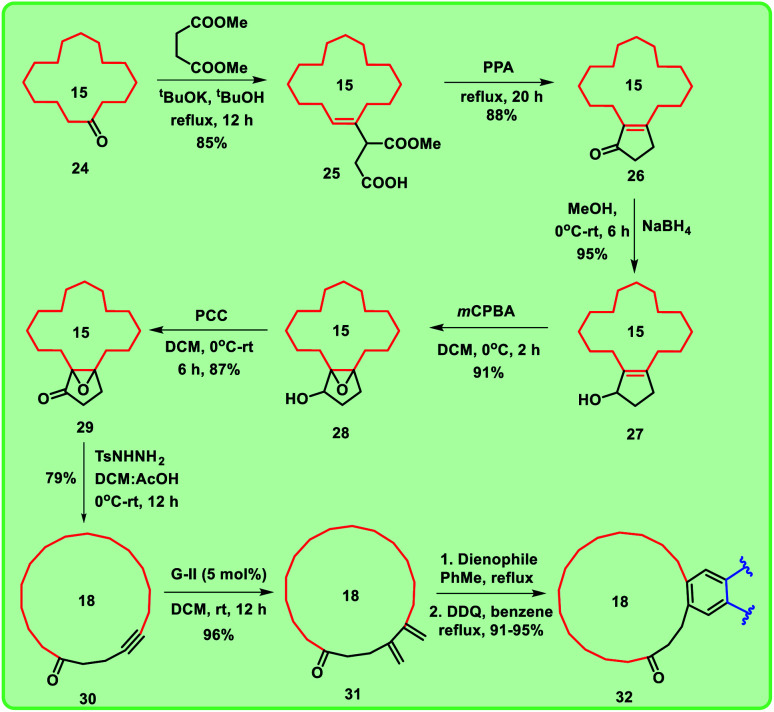
Synthesis of mixed musks from cyclopentadecanone.

**Fig. 2:**
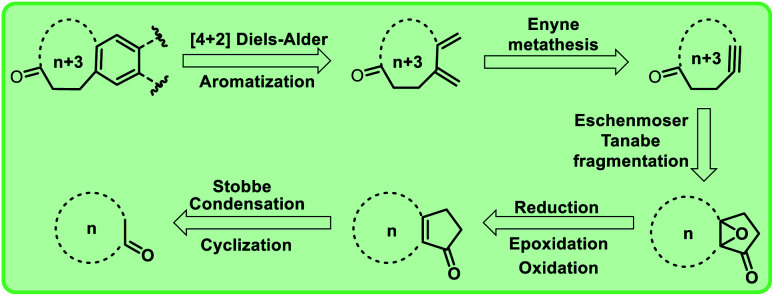
Retrosynthetic route to macrocyclic musk analogues.

In view of the importance of macrocyclic ketones as well as our long-term interest in olefin metathesis and other strategies to design polycycles,^8^ we conceived a new synthetic approach (Schemes 1–5) to mixed musks containing fused cyclic ketone derivatives based on cross-enyne metathesis as key step. Retrosynthetic analysis to these macrocyclic compounds is shown in Fig. 2.

## Results and discussion

Considering the importance of macrocyclic ring systems in designing musks, we started with commercially available cyclododecanone 13. The compound 13 underwent Stobbe condensation with dimethyl succinate under basic conditions to produce the compound 14 in good yield (88%).

Further, compound 14 was treated with polyphosphoric acid (PPA) at 95–100 °C for 3 h, we obtained cyclopentane derivative 15 as major product with the corresponding decarboxylated product 16.^9^ Under acidic conditions, the mixture 15 and 16 collectively converted into decarboxylated enone 16.

Alternatively, when the compound 14 was heated with PPA for 20 h, enone 16 was formed exclusively (Scheme 1). To obtain the key intermediate 19, compound 16 was treated with hydrogen peroxide (H_2_O_2_) under different reaction conditions at room temperature in the presence of bases like NaOH and K_2_CO_3_ for 12 h. Unfortunately, trace amount of keto-epoxide 19 was observed. In this regard, to get higher yield of the compound 19 from enone 16, alternate method was adopted in which enone 16 was initially reduced to hydroxy derivative 17 using sodium borohydride (NaBH_4_). Compound 17 was then treated with *m*CPBA to give hydroxy-epoxide 18 which further on reaction with pyridinium chlorochromate (PCC) produced the oxidised keto-epoxide 19 (Scheme 2). It is worthy to mention, by using this alternate route, we obtained the desired key intermediate 19 in good yield.

Further, we are intended to synthesize a metathesis precursor *i.e.* macrocyclic alkyne 21 using compound 19. In view of this aspect, compound 19 was treated with tosyl hydrazide in ethanol under reflux conditions to afford the compound 20, which subsequently on reaction with 1,3-dibromo-5,5-dimethylhydantoin in isopropyl alcohol (IPA) delivered the corresponding rearranged 15-membered macrocyclic derivative 21 in very low yield (5%). This problem was rectified by using the key reaction Eschenmoser–Tanabe fragmentation.^10^

To access enlarged 15-membered macrocyclic compound 21 in good yield, compound 19 was directly treated with tosyl hydrazide in the presence of DCM:AcOH mixture (Scheme 3).

To prepare the diene, cross-enyne metathesis^11^ was performed on compound 21 using Grubbs II generation catalyst and the diene 22 was formed in excellent yield, which is a useful substrate for the Diels–Alder reaction. To access the targeted fused macrocyclic systems, the macrocyclic diene 22 was subjected to Diels–Alder reaction^12^ followed by aromatization with 2,3-dichloro-5,6-dicyano-1,4-benzoquinone (DDQ). Here, we used different dienophiles to realize [4 + 2] cycloaddition products to generate 15 membered mixed musks (Scheme 4).

**Fig. 3:**
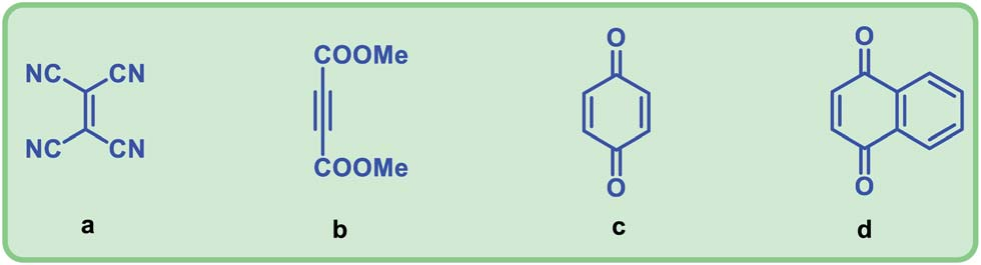
Dienophiles used in the synthesis of mixed musks.

We have used four types of dienophiles here such as tetracyanoethylene (a), dimethyl acetylenedicarboxylate (b), benzoquinone (c) and naphthoquinone (d) to assemble mixed musks having 15 membered macrocycle (Fig. 3).

**Fig. 4:**
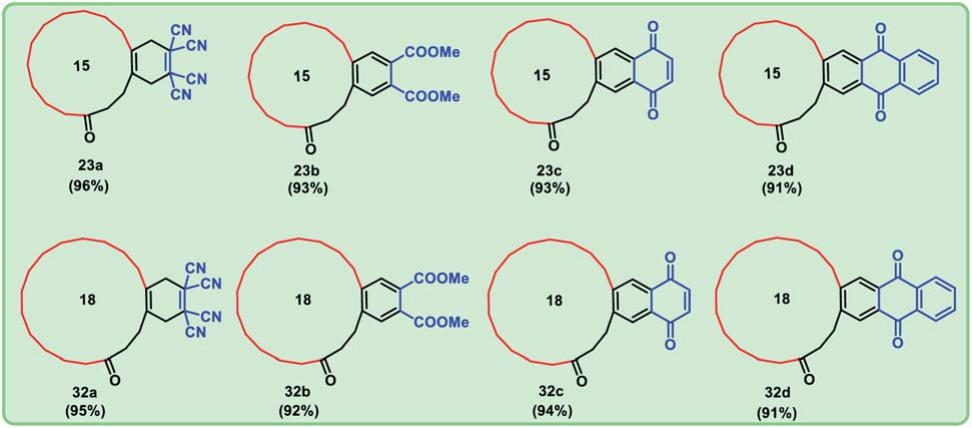
Different macrocycles prepared in our study along with the last step yields.

Using these optimized conditions, we have carried out the sequence with commercially available cyclopentadecanone 24, which depicted in Scheme 5. By using a similar strategy, we successfully synthesized four different mixed musks in good yields with the difference in macrocyclic ring system. Thus, we obtained eight targeted mixed musks (Fig. 4).

## Conclusions

We have successfully demonstrated a new synthetic strategy to prepare mixed musks having macrocyclic ring systems fused with polycycles in good yields. This route includes the usage of commercially available materials and facile reaction conditions. In targeted molecules (Fig. 4), 15-membered and 18-membered macrocyclic systems are shown here, as the difference in ring system can lead to a change in the properties of musks. This strategy may be valuable addition to the flavor and fragrance industry.

## Conflicts of interest

There are no conflicts to declare.

## Supplementary Material

RA-012-D2RA01458K-s001
