# Supplementary material for: Synthesis of mixed musks via Eschenmoser–Tanabe fragmentation, enyne metathesis and Diels–Alder reaction as key steps
Source: RSC Adv. 2022 May 11;12(22):14278–81. doi: 10.1039/d2ra01458k (PMC9092383; doi:10.1039/d2ra01458k)

Electronic Supplementary Material (ESI) for RSC Advances.
This journal is © The Royal Society of Chemistry 2022

Synthesis of mixed musks via Eschenmoser-Tanabe fragmentation, enyne metathesis and Diels-Alder reaction as key steps

Sambasivarao Kotha,[a*] Arpit Agrawal [a†] and Yellaiah Tangella [a†]

Department of Chemistry, Indian Institute of Technology, Bombay,

Powai, Mumbai, Maharashtra, India-400076

E-mail: srk@chem.iitb.ac.in

Table of Contents

1. General Experimental Procedures and Characterization Data…………… .2- 12
2. ^{1}H-NMR and ^{13}C-NMR Spectra…………………………………………...13 – 34

GENERAL EXPERIMENTAL PROCEDURES AND CHARACTERIZATION DATA

General Information

All the reactions were monitored by thin-layer chromatography (TLC) using appropriate solvent systems. Column chromatography was performed by using Acme's silica gel (100–200 mesh) with an appropriate mixture of EtOAc and petroleum ether. Yields refer to samples which are chromatographically separated. Without further purification, all the commercial grade reagents were used. In general, NMR samples have been analysed in $CDCl_3$ solvent and chemical changes are recorded as an internal standard in δ values using tetramethylsilane (TMS). The standard abbreviations for singlet, doublet, triplet, doublet of doublet and multiplet are: s, d, t, dd, and m. The constants of coupling (*J*) are recorded in Hz. Bruker (AVANCE IIITM) 500 MHz and Bruker (AVANCE IIITM) 400 MHz spectrometers were used to record both ^{1}H NMR and ^{13}C NMR spectroscopic data. The high-resolution mass measurements were carried out by using an electrospray ionization (ESI) spectrometer. Melting points were recorded on a Veego melting point apparatus.

Synthesis of (E)-3-(cyclododec-1-en-1-yl)-4-methoxy-4-oxobutanoic acid (14)

To the mixture of cyclododecanone (10g, 54.8 mmol) and dimethyl succinate (DMS) (9.61g, 65.8 mmol) in ^{t}BuOH (100 ml) was added ^{t}BuOK (7.38g, 65.8 mmol) under nitrogen and the reaction mixture stirred at reflux for 20 h. After monitoring via TLC, reaction mixture was cooled to rt and ^{t}BuOH was removed under vacuum. The crude was acidified with 6N HCl solution (ph=2-3). Then the reaction mixture was extracted 3 times with ether. Now the organic layer washed with water. Later, ether layer was extracted with 1.7N Ammonia solution. Then this ammonia layer was acidified with 50% HCl solution (ph=2-3) and extracted 3 times with ether. The combined organic layer dried over sodium sulphate and concentrated under reduced pressure. The crude product was purified by column chromatography on silica gel, eluting with 10% ethyl acetate in pet ether to get pure product **14**.

Pure yield of **14**: 14.3g, 88%; Brown liquid

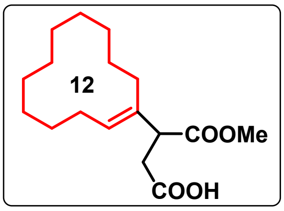

^{1}H NMR (500 MHz, $CDCl_3$): δ_H 5.31(t, *J*=7.8 Hz, 1H), 3.68(s, 3H), 3.44-3.41(m, 1H), 3.01-2.95(m, 1H), 2.52(dd, *J*=4.63,12.48 Hz, 1H), 2.18(t, *J*=6.74 Hz, 2H), 2.09-2.01(m, 2H), 1.43-1.33(m, 16H); **^{13}C NMR** (125 MHz, $CDCl_3$): δ_C 178.0, 174.2, 135.7, 129.9, 52.2, 45.5,

36.5, 27.0, 26.9, 25.4, 25.1, 25.0, 24.8, 24.6, 24.0, 22.9, 22.3; **HRMS** (ESI) m/z calcd. for $C_{17}H_{28}O_4Na[M + Na]^+$: 319.1877; found: 319.1877

Synthesis of (E)-3-(cyclopentadec-1-en-1-yl)-4-methoxy-4-oxobutanoic acid (25)

To the mixture of cyclopentadecanone (5g, 22.28 mmol) and dimethyl succinate (DMS) (2.71g, 18.56 mmol) in ^{t}BuOH (50 ml) was added ^{t}BuOK (2.08g, 18.56 mmol) under nitrogen and the reaction mixture stirred at reflux for 20 h. After monitoring via TLC, reaction mixture was cooled to rt and ^{t}BuOH was removed under vacuum. The crude was acidified with 6N HCl solution (ph=2-3). Then the reaction mixture was extracted 3 times with ether. Now the organic layer washed with water. Later, ether layer was extracted with 1.7N Ammonia solution. Then this ammonia layer was acidified with 50% HCl solution (ph=2-3) and extracted 3 times with ether. The combined organic layer dried over sodium sulphate and concentrated under reduced pressure. The crude product was purified by column chromatography on silica gel, eluting with 10% ethyl acetate in pet ether to get pure product **25**.

Pure yield of **25**: 6.40g, 85%; Pale yellow solid; M.P. 72-74 °C

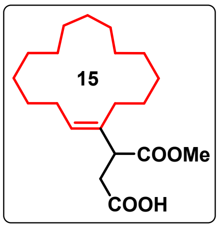

^{1}H NMR (400 MHz, $CDCl_3$): δ_H 10.19 (bs, 1H), 5.32 (t, *J*=7.42 Hz, 1H), 3.66 (s, 3H), 3.42-3.38 (m, 1H), 2.99-2.92 (m, 1H), 2.49 (dd, *J*=17.3, 4.8 Hz, 1H), 2.10-1.97 (m, 4H), 1.39-1.22 (m, 22H); **^{13}C NMR** (100 MHz, $CDCl_3$): δ_C 178.1, 173.9, 135.8, 129.6, 52.1, 47.3, 36.3, 29.9, 27.9, 27.7, 27.5, 27.4 ,27.3, 27.1, 26.9, 26.7, 26.4, 26.3, 26.0, 25.9; **HRMS** (ESI) m/z calcd. for $C_{20}H_{34}O_4Na[M + Na]^+$: 361.2358; found: 361.2357.

Synthesis of methyl 3-oxo-2,3,4,5,6,7,8,9,10,11,12,13-dodecahydro-1*H*-cyclopenta[12]annulene-1-carboxylate (15)

Polyphosphoric Acid (10 ml) was taken in RB flask and heated to reach 95-100°C and then the hot PPA was added to compound **14** (1g, 3.3 mmol) in another RB flask. Then this reaction mixture heated to 95-100 °C for 3 h. After completion, the reaction mixture was cooled to rt and ice-cold water was added add stirred for 5-10 min. Aqueous layer was extracted 3 times with ether and combined organic layer washed with water and dried over sodium sulphate and concentrated under reduced pressure to afford crude product. The crude product was purified by column chromatography on silica gel, eluting with 10% ethyl acetate in pet ether to get pure product **15** and **16**.

Pure yield of **15**: 516mg, 55%; Brown gel

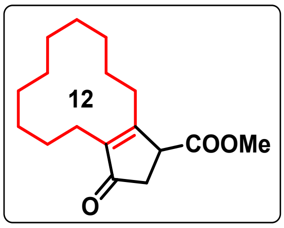

^{1}H NMR (500 MHz, $CDCl_3$): δ_H 3.72(s, 3H), 2.73-2.67(m, 1H), 2.63-2.54(m, 2H), 2.31-2.12(m, 3H), 1.82-1.74(m, 1H), 1.61-1.53(m, 4H), 1.44-1.18(m, 12H); **^{13}C NMR** (125 MHz, $CDCl_3$): δ_C 207.8, 173.1, 169.8, 142.0, 52.5, 46.0, 38.5, 26.4, 25.7, 25.6, 25.4, 24.7, 24.6, 23.4, 23.0, 21.8, 21.1; **HRMS** (ESI) m/z calcd. for $C_{17}H_{26}O_3Na[M + Na]^+$: 301.1774; found: 301.1777.

Pure yield of **16**: 111mg, 15%; Brown liquid

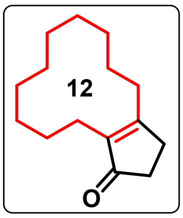

^{1}H NMR (500 MHz, $CDCl_3$): δ_H 2.49-2.48(m, 2H), 2.43(t, *J*=7.46 Hz, 2H), 2.36-2.34(m, 2H), 2.23(t, *J*=6.71 Hz, 2H), 1.69-1.64(m, 2H), 1.60-1.58 (m, 2H), 1.46-1.32(m, 10H), 1.17-1.12(m, 2H); **^{13}C NMR** (125 MHz, $CDCl_3$): δ_C 210.7, 174.7, 140.1, 34.4, 29.0, 28.1, 25.7, 25.6, 25.3, 24.9, 24.7, 23.6, 22.9, 21.9, 20.7; **HRMS** (ESI) m/z calcd. for $C_{15}H_{24}ONa[M + Na]^+$: 243.1718; found: 243.1718.

Synthesis of 2,3,4,5,6,7,8,9,10,11,12,13-dodecahydro-1*H*-cyclopenta[12]annulen-1-one (16)

Polyphosphoric Acid (10 ml) was taken in RB flask and heated to reach 95-100 °C and then the hot PPA was added to compound **14** (5g, 16.8 mmol) in another RB flask. Then this reaction mixture heated to 95-100 °C for 20 h. After completion, the reaction mixture was cooled to rt and ice-cold water was added add stirred for 5-10 min. Aqueous layer was extracted 3 times with ether and combined organic layer washed with water and dried over sodium sulphate and concentrated under reduced pressure to afford crude product. The crude product was purified by column chromatography on silica gel, eluting with 10% ethyl acetate in pet ether to get pure product **16**.

Pure yield of **16**: 3.3g, 89%; Brown liquid

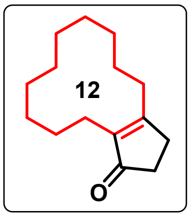

^{1}H NMR (500 MHz, $CDCl_3$): δ_H 2.49-2.48(m, 2H), 2.43(t, *J*=7.46 Hz, 2H), 2.36-2.34(m, 2H), 2.23(t, *J*=6.71 Hz, 2H), 1.69-1.64(m, 2H), 1.60-1.58(m, 2H), 1.46-1.32(m, 10H), 1.17-1.12(m, 2H); **^{13}C NMR** (125 MHz, $CDCl_3$): δ_C 210.7, 174.7, 140.1, 34.4, 29.0, 28.1, 25.7, 25.6, 25.3, 24.9, 24.7, 23.6, 22.9, 21.9, 20.7; **HRMS** (ESI) m/z calcd. for $C_{15}H_{24}ONa[M + Na]^+$: 243.1718; found: 243.1718.

Synthesis of 3,4,5,6,7,8,9,10,11,12,13,14,15,16-tetradecahydrocyclopenta[15]annulen-1(2*H*)-one (26)

Polyphosphoric Acid (10 ml) was taken in RB flask and heated to reach 95-100 °C and then the hot PPA was added to compound **25** (6g, 17.73 mmol) in another RB flask. Then this reaction mixture heated to 95-100 °C for 20 h. After completion, the reaction mixture was cooled to rt and ice-cold water was added add stirred for 5-10 min. Aqueous layer was extracted 3 times with ether and combined organic layer washed with water and dried over sodium sulphate and concentrated under reduced pressure to afford crude product. The crude product was purified by column chromatography on silica gel, eluting with 10% ethyl acetate in pet ether to get pure product **26**.

Pure yield of **26**: 4.09g, 88%; Yellow liquid

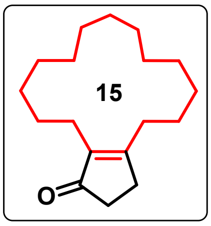

^{1}H NMR (400 MHz, $CDCl_3$): δ_H 2.47-2.45 (m, 2H), 2.37-2.31 (m, 4H), 2.12-2.10 (m, 2H), 1.56-1.49 (m, 2H), 1.46-1.22 (m, 20H); **^{13}C NMR** (100 MHz, $CDCl_3$): δ_C 210.2, 174.4, 140.5, 34.4, 31.2, 29.5, 27.7, 27.6, 27.2, 27.0, 26.9, 26.3, 26.1, 26.0, 25.8, 25.6, 22.9; **HRMS** (ESI) m/z calcd. for $C_{18}H_{30}ONa[M + Na]^+$: 285.2184; found: 285.2184.

Synthesis of 2,3,4,5,6,7,8,9,10,11,12,13-dodecahydro-1*H*-cyclopenta[12]annulen-1-ol (17)

Compound **16** (3g, 13.6 mmol) was dissolved in methanol (30 ml) and sodium borohydride (1g, 27.2 mmol) was added portion wise at 0 °C and stirred at rt for 3 h. After 3 h, reaction quenched with sat. Ammonium chloride solution and then methanol was removed under pressure. Then, aq. phase extracted 3 times with chloroform. The combined organic layer was concentrated and the crude compounds **17** were directly used for next step without further purification.

Crude yield of **17**: 2.7g, 92%

Synthesis of 1,2,3,4,5,6,7,8,9,10,11,12,13,14,15,16-hexadecahydrocyclopenta[15]annulen-1-ol (27)

Compound **26** (4g, 15.25 mmol) was dissolved in methanol (40 ml) and sodium borohydride (1.16g, 30.5 mmol) was added portion wise at 0 °C and stirred at rt for 3 h. After 3 h, reaction quenched with sat. Ammonium chloride solution and then methanol was removed under pressure. Then, aq. phase extracted 3 times with chloroform. The combined organic layer was concentrated and the crude compounds **27** were directly used for next step without further purification.

Crude yield of **27**: 3.8g, 95%

Synthesis of dodecahydro-1*H*-3a,13a-epoxycyclopenta[12]annulen-1-ol (18)

To a stirred solution of compound **17** (2g, 9 mmol) in DCM (30 ml), was added m-CPBA (2.3g, 13.5 mmol) portion wise slowly at 0 °C under nitrogen atmosphere. The reaction mixture was stirred without removal of ice bath for 2 h. Then quenched with saturated sodium bicarbonate solution. The aq. layer extracted 3 times with DCM. The combined organic layer washed with saturated sodium bicarbonate solution. Then organic layer was dried over sodium sulphate and concentrated under reduced pressure. The crude product was purified by column chromatography on silica gel, eluting with 10% ethyl acetate in pet ether to get pure product **18**.

Pure yield of **18**: 1.9g, 90%; White solid; M.P. 88-90 °C

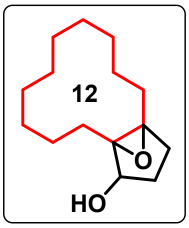

^{1}H NMR (500 MHz, $CDCl_3$): δ_H 4.15(t, *J*=7.96 Hz, 1H), 2.06-2.00(m, 1H), 1.94-1.89(m, 1H), 1.82-1.64(m, 6H), 1.55-1.49(m, 4H), 1.42-1.32(m, 9H), 1.26-1.20(m, 4H); **^{13}C NMR** (125 MHz, $CDCl_3$): δ_C 73.2, 72.7, 71.3, 27.8, 27.3, 27.1, 26.7, 26.6, 26.2, 26.1, 23.7, 23.6, 23.2, 22.45, 22.42; **HRMS** (ESI) m/z calcd. for $C_{15}H_{27}O_2[M + H]^+$: 239.2004; found: 239.2003.

Synthesis of tetradecahydro-1*H*,4*H*-3a,16a-epoxycyclopenta[15]annulen-1-ol (28)

To a stirred solution of compound **27** (3.5g, 13.24 mmol) in DCM (40 ml), was added m-CPBA (3.41g, 19.86 mmol) portion wise slowly at 0 °C under nitrogen atmosphere. The reaction mixture was stirred without removal of ice bath for 2 h. Then quenched with saturated sodium bicarbonate solution. The aq. layer extracted 3 times with DCM. The combined organic layer washed with saturated sodium bicarbonate solution. Then organic layer was dried over sodium sulphate and concentrated under reduced pressure. The crude product was purified by column chromatography on silica gel, eluting with 10% ethyl acetate in pet ether to get pure product **28**.

Pure yield of **28**: 3.37g, 91%; White solid; M.P. 68-70 °C

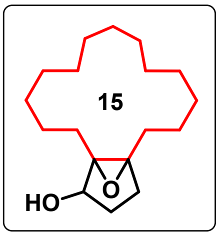

^{1}H NMR (400 MHz, $CDCl_3$): δ_H 4.13 (t, *J*=7.90 Hz, 1H), 2.06-1.16 (m, 31H); **^{13}C NMR** (100 MHz, $CDCl_3$): δ_C 74.0, 72.1, 70.7, 30.5, 38.1, 27.7, 27.4, 27.3, 26.9, 26.5, 25.9, 25.8, 25.4, 25.2, 24.2, 23.7; **HRMS** (ESI) m/z calcd. for $C_{18}H_{33}O_2[M + H]^+$: 281.2477; found: 281.2477.

Synthesis of dodecahydro-1*H*-3a,13a-epoxycyclopenta[12]annulen-1-one (19)

To a stirred solution of compound **18** (1.5g, 6.2 mmol) in DCM (30 ml), PCC (2.7g, 12.5 mmol) was added portion wise at 0 °C and stirred at rt for 6 h. After completion of the reaction, the solvent was removed and The crude product was purified by column chromatography on silica gel, eluting with 5% ethyl acetate in pet ether to get pure product **19**.

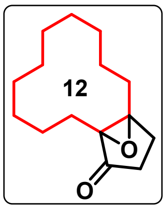

Pure yield of **19**: 1.2g, 81%; Colourless liquid

^{1}H NMR (500 MHz, $CDCl_3$): δ_H 2.36-2.28 (m, 1H), 2.11-2.03 (m, 4H), 1.98-1.93 (m, 1H), 1.77-1.61 (m, 4H), 1.51-1.26 (m, 14H); **^{13}C NMR** (125 MHz, $CDCl_3$): δ_C 212.1, 73.4, 68.5, 32.0, 27.6, 27.0, 26.8, 26.7, 26.1, 24.6, 23.6, 23.4, 22.4, 22.2, 21.8; **HRMS** (ESI) m/z calcd. for $C_{15}H_{25}O_2[M + H]^+$: 237.1852; found: 237.1852.

Synthesis of tetradecahydro -1*H*,4*H*-3a,16a-epoxycyclopenta[15]annulen-1-one (29)

To a stirred solution of compound **28** (3g, 10.7 mmol) in DCM (50 ml), PCC (4.6g, 21.4 mmol) was added portion wise at 0 °C and stirred at rt for 6 h. After completion of the reaction, the solvent was removed and The crude product was purified by column chromatography on silica gel, eluting with 5% ethyl acetate in pet ether to get pure product **29**.

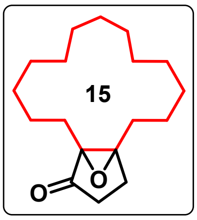

Pure yield of **29**: 2.59g, 87%; Pale yellow liquid

^{1}H NMR (400 MHz, $CDCl_3$): δ_H 2.27-2.18 (m, 1H), 2.09-2.04 (m, 1H), 1.99-1.76 (m, 4H), 1.62-1.54 (m, 1H), 1.51-1.19 (m, 23H); **^{13}C NMR** (100 MHz, $CDCl_3$): δ_C 211.9, 72.1, 67.2, 31.7, 30.0, 26.9, 26.8, 26.6, 26.4, 26.3, 25.7, 25.6, 25.3, 25.2, 24.9, 23.6, 23.1, 22.7; **HRMS** (ESI) m/z calcd. for $C_{18}H_{31}O_2[M + H]^+$: 279.2319; found: 279.2318.

Synthesis of (*E*)-4-methyl-*N*'-(tetradecahydro-1*H*-cyclopenta[12]annulen-1-ylidene)benzene sulfonohydrazide(20)

To a stirred solution of compound **19** (200mg, 0.8mmol) in dry ethanol (15 ml) was added 4-methylbenzenesulfonohydrazide (236mg, 1.2 mmol) under nitrogen atmosphere. The reaction mixture was stirred under reflux temperature for 3 h. After completion of the reaction by TLC, half of the ethanol was removed *in vacuo*. Then the reaction mixture cooled to 0 °C and stirred for 5-10 min. The obtained solid was filtered and washed with minimum amount of cold ethanol and dried under *vacuum*.

Pure yield of **20**: 260mg, 76%; White solid; M.P. 169-171 °C

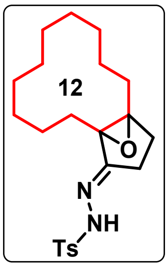

^{1}H NMR (500 MHz, $CDCl_3$): δ_H 7.89(d, *J*=8.22 Hz, 2H), 7.31-7.28(m, 3H), 2.43-2.40(m, 7H), 2.29-2.26(m, 4H), 1.55-1.53(m, 4H), 1.43-1.29(m, 10H), 1.10-1.09(m, 2H); **^{13}C NMR** (125 MHz, $CDCl_3$) : δ_C 143.8, 136.3, 135.6, 129.4, 128.3, 31.5, 26.6, 25.3, 25.2, 25.18, 25.13, 25.12, 24.9, 23.7, 22.8, 21.9, 21.7, 21.4; **HRMS** (ESI) m/z calcd. for $C_{22}H_{32}N_2O_3S[M + Na]^+$: 427.2026; found: 427.2028.

Synthesis of cyclopentadec-4-yn-1-one (21)

To a stirred solution of compound **19** (700mg, 2.9 mmol) in a 1:1 mixture of DCM:AcOH (10 ml : 10 ml), tosyl hydrazide (662mg, 3.5 mmol) was added at 0°C and stirred at 0°C for 45 min. The reaction mixture slowly warmed to rt and stirred for 12 h. Then, AcOH was removed and the crude mixture was basified with saturated sodium carbonate solution. Then, the reaction mixture was extracted 3 times with ether. The combined organic layer dried over sodium sulphate and concentrated under reduced pressure to get crude compound. The crude product was purified by column chromatography on silica gel, eluting with 5% ethyl acetate in pet ether to get pure product **21**.

Pure yield of **21**: 489mg, 75%; Pale brown wet solid

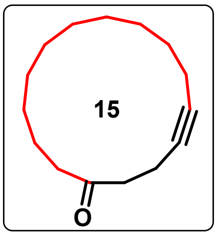

^{1}H NMR (500 MHz, $CDCl_3$): δ_H 2.54-2.45 (m, 6H), 2.18-2.17 (m, 2H), 1.70-1.64 (m, 2H), 1.41-1.26 (m, 14H); **^{13}C NMR** (125 MHz, $CDCl_3$): δ_C 210.5, 80.9, 79.2, 42.3, 41.6, 28.1, 27.3, 27.1, 26.5, 26.1, 25.5, 25.2, 21.2, 18.2, 14.7; **HRMS** (ESI) m/z calcd. for $C_{15}H_{25}O[M + H]^+$: 221.1901; found: 221.1901.

Synthesis of cyclooctadec-4-yn-1-one (30)

To a stirred solution of compound **29** (2g, 7.1 mmol) in a 1:1 mixture of DCM:AcOH (20 ml : 20 ml), tosyl hydrazide (1.6g, 8.6 mmol) was added at 0°C and stirred at 0°C for 45 min. The reaction mixture slowly warmed to rt and stirred for 12 h. Then, AcOH was removed and the crude mixture was basified with saturated sodium carbonate solution. Then, the reaction mixture was extracted 3 times with ether. The combined organic layer dried over sodium sulphate and concentrated under reduced pressure to get crude compound. The crude product

was purified by column chromatography on silica gel, eluting with 5% ethyl acetate in pet ether to get pure product **30**.

Pure yield of **30**: 1.48g, 79%; Colourless liquid.

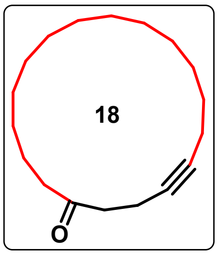

^{1}H NMR (400 MHz, $CDCl_3$): δ_H 2.50 (t, *J*=6.32 Hz, 2H), 2.35-2.28 (m, 4H), 2.03-2.00 (m, 2H), 1.56-1.49 (m, 2H), 1.35-1.15 (m, 20H); **^{13}C NMR** (100 MHz, $CDCl_3$): δ_C 209.3, 80.6, 78.9, 42.8, 41.4, 28.2, 27.8, 27.5, 27.48, 27.42, 27.40, 27.3, 26.8, 26.6, 26.5, 23.0, 18.4, 13.5; **HRMS** (ESI) m/z calcd. for $C_{18}H_{31}O[M + H]^+$: 263.2374; found: 263.2373.

Synthesis of 4,5-dimethylenecyclopentadecan-1-one (22)

G-II (5 mol%) was added to a stirred degassed solution of compound **21** (400mg, 1.8 mmol) in dry DCM (10 ml) at rt. The reaction mixture was stirred for 10 h under ethylene atmosphere. After completion of the reaction, the reaction mixture was concentrated and the crude product was purified by column chromatography on silica gel, eluting with 2% ethyl acetate in pet ether to get pure product **22**.

Pure yield of **22**: 428mg, 95%; Pale yellow liquid

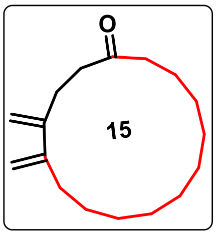

^{1}H NMR (400 MHz, $CDCl_3$): δ_H 5.09-4.96 (m, 4H), 2.62-2.54 (m, 4H), 2.32-2.26 (m, 4H), 1.61-1.54 (m, 2H), 1.44-1.37 (m, 2H), 1.31-1.25 (m, 12H); **^{13}C NMR** (100 MHz, $CDCl_3$): δ_C 212.1, 146.8, 146.1, 113.8, 112.9, 42.6, 41.4, 34.3, 28.8, 27.6, 27.08, 27.05, 26.8, 26.5, 25.9, 25.8, 22.9; **HRMS** (ESI) m/z calcd. for $C_{17}H_{29}O[M + H]^+$: 249.2209; found: 249.2209.

Synthesis of 4,5-dimethylenecyclooctadecan-1-one (31)

G-II (5 mol%) was added to a stirred degassed solution of compound **30** (500mg, 1.9 mmol) in dry DCM (10 ml) at rt. The reaction mixture was stirred for 10 h under ethylene atmosphere. After completion of the reaction, the reaction mixture was concentrated and the crude product was purified by column chromatography on silica gel, eluting with 2% ethyl acetate in pet ether to get pure product **31**.

Pure yield of **31**: 530mg, 96%; Colourless liquid

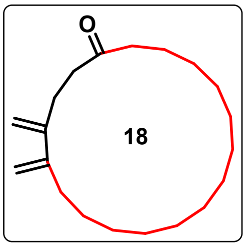

^{1}H NMR (500 MHz, $CDCl_3$): δ_H 5.07-4.97 (m, 4H), 2.58 (s, 4H), 2.36 (t, *J*=7.22 Hz, 2H), 2.28 (t, *J*=7.07 Hz, 2H), 1.60-1.57 (m, 2H), 1.44-1.41 (m,

2H), 1.28 (s, 18H); **^{13}C NMR** (125 MHz, $CDCl_3$): δ_C 211.3, 147.1, 146.1, 112.9, 112.3, 43.0, 41.2, 34.4, 28.6, 28.3, 28.2, 27.6, 27.56, 27.54, 27.44, 27.41, 27.3, 26.9, 26.8, 23.6; **HRMS** (ESI) m/z calcd. for $C_{20}H_{34}O[M + H]^+$: 291.2699; found: 291.2699.

General procedure for the synthesis of 23a-23d and 32a-32d

To a stirred solution of compound **22/31** (50mg) in aniline, dienophile **a-d** (1.2 eq.) [tetracyanoethylene (**a**), dimethyl acetylenedicarboxylate (**b**), benzoquinone (**c**) and naphthoquinone (**d**)] was added at rt under inert atmosphere and the reaction mixture was refluxed for 12 h. Then solvent was removed under reduced pressure to get crude product. Without further purification, the crude product was dissolved in benzene and DDQ (1.5 eq) was added in one portion and reflux it for 6 h. Now the reaction mixture was cooled to rt and basic $Na_2S_2O_3$ solution (10% $Na_2S_2O_3$ and 10% NaOH) was added and the mixture was stirred for another 5 min. The aqueous phase was extracted 3 times with diethyl ether, washed with brine, and dried over anhydrous sodium sulphate. The crude product was purified by column chromatography on silica gel, eluting with 10% ethyl acetate in pet ether to get pure product.

Pure yield of **23a**: 72mg, 96%; White solid; M.P. 136-138 °C

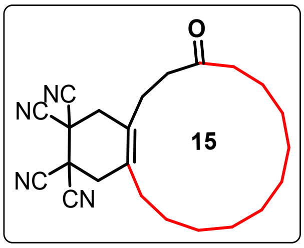

^{1}H NMR (400 MHz, $CDCl_3$): δ_H 3.09 (s, 2H), 3.03 (s, 2H), 2.58-2.54 (m, 2H), 2.43-2.37 (m, 4H), 2.12-2.09 (m, 2H), 1.70-1.63 (m, 2H), 1.43-1.26 (m, 14H); **^{13}C NMR** (100 MHz, $CDCl_3$): δ_C 210.3, 127.8, 125.4, 110.8, 110.7, 42.7, 40.4, 38.06, 38.02, 36.0, 31.5, 27.2, 26.79, 26.74, 26.64, 26.60, 26.17, 26.14, 25.5, 24.0; **HRMS** (ESI) m/z calcd. for $C_{23}H_{28}N_4ONa[M + Na]^+$: 399.2156; found: 399.2156.

Pure yield of **23b**: 72mg, 93%; White Solid; M.P. 101-103 °C

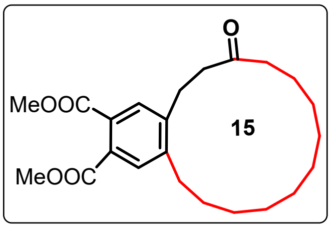

^{1}H NMR (400 MHz, $CDCl_3$): δ_H 7.49 (s, 2H), 3.87 (s, 6H), 2.95-2.90 (m, 2H), 2.71-2.67 (m, 2H), 2.62-2.58 (m, 2H), 2.42-2.39 (m, 2H), 1.69-1.54 (m, 4H), 1.50-1.43 (m, 2H), 1.39-1.23 (m, 10H); **^{13}C NMR** (100 MHz, $CDCl_3$): δ_C 210.8, 168.3, 168.2, 144.6, 142.7, 130.3, 130.2, 130.1, 129.7, 52.7, 52.4, 43.9, 42.6, 32.3, 29.8, 29.2, 27.4, 27.1, 26.8, 26.7, 26.4, 25.5, 24.2; **HRMS** (ESI) m/z calcd. for $C_{23}H_{32}O_5Na[M + Na]^+$: 411.2142; found: 411.2142.

Pure yield of **23c**: 66mg, 93%; Yellow Solid; M.P. 133-135 °C

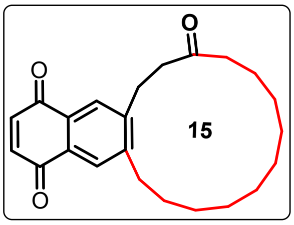

^{1}H NMR (400 MHz, $CDCl_3$): δ_H 7.82 (d, *J*=6.90 Hz, 2H), 6.88 (s, 2H), 3.01-2.97 (m, 2H), 2.74-2.65 (m, 4H), 2.41 (t, *J*=6.29 Hz, 2H), 1.67-1.57 (m, 4H), 1.50-1.46 (m, 2H), 1.37-1.27 (m, 10H); **^{13}C NMR** (100 MHz, $CDCl_3$): δ_C 210.5, 185.3, 185.1, 147.9, 146.2, 138.8, 130.3, 130.1, 127.7, 127.6, 43.7, 42.5, 32.6, 29.1, 27.4, 27.3, 26.8, 26.7, 26.4, 26.3, 25.5, 24.2; **HRMS** (ESI) m/z calcd. for $C_{23}H_{29}O_3[M + H]^+$: 375.1934; found: 375.1934.

Pure yield of **23d**: 73 mg, 91%; White solid; M.P. 197-199 °C

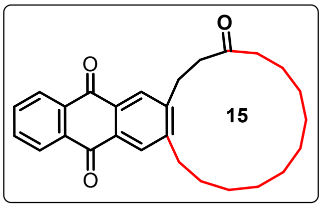

^{1}H NMR (500 MHz, $CDCl_3$): δ_H 8.28-8.27 (m, 2H), 8.06 (d, *J*=8.99 Hz, 2H), 7.78-7.76 (m, 2H), 3.06-3.03 (m, 2H), 2.79-2.70 (m, 4H), 2.44 (t, *J*=6.36 Hz, 2H), 1.71-1.63 (m, 5H), 1.55-1.49 (m, 2H), 1.41-1.29 (m, 9H); **^{13}C NMR** (125 MHz, $CDCl_3$): δ_C 211.7, 183.3, 183.2, 148.3, 146.5, 134.14, 134.13, 133.8, 133.7, 131.9, 131.8, 128.5, 128.4, 127.3, 43.9, 42.6, 32.7, 29.2, 27.5, 27.4, 26.8, 26.7, 26.4, 26.3, 25.6, 24.3; **HRMS** (ESI) m/z calcd. for $C_{27}H_{31}O_3[M + H]^+$: 425.2090; found: 425.2090.

Pure yield of **32a**: 68mg, 95%; Pale brown solid; M.P. 147-149 °C

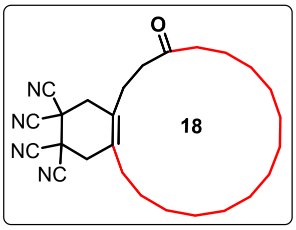

^{1}H NMR (500 MHz, $CDCl_3$): δ_H 3.06 (s, 2H), 3.02 (s, 2H), 2.53-2.50 (m, 2H), 2.43-2.38 (m, 4H), 2.14 (t, *J*=7.52 Hz, 2H), 1.65-1.61 (m, 2H), 1.42-1.40 (m, 2H), 1.31-1.28 (m, 18H); **^{13}C NMR** (125 MHz, $CDCl_3$): δ_C 209.2, 127.7, 125.3, 110.83, 110.80, 42.2, 40.8, 38.0, 36.2, 35.8, 32.0, 28.5, 28.1, 28.0, 27.5, 27.4, 27.3, 26.96, 26.91, 26.7, 26.6, 26.0, 23.3; **HRMS** (ESI) m/z calcd. for $C_{26}H_{35}N_4O[M + H]+$: 419.2805; found: 419.2805.

Pure yield of **32b**: 68mg, 92%; White Solid; M.P. 71-73 °C

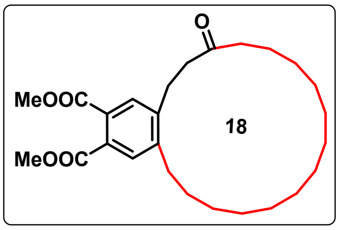

^{1}H NMR (500 MHz, $CDCl_3$): δ_H 7.48 (d, *J*=11.89 Hz, 2H), 3.87 (d, *J*=1.96 Hz, 6H), 2.93-2.90 (m, 2H), 2.68-2.61 (m, 4H), 2.43 (t, *J*=6.61 Hz, 2H), 1.65-1.55 (m, 4H), 1.40-1.26 (m, 18H); **^{13}C NMR** (125 MHz, $CDCl_3$): δ_C 209.8, 168.3, 168.2, 144.7, 142.5, 130.2, 130.1, 129.9, 129.6, 52.6, 44.0, 42.0, 32.5, 31.1, 29.0, 27.99, 27.94, 27.7, 27.6, 27.1, 27.0, 26.8, 26.7, 23.4; **HRMS** (ESI) m/z calcd. for $C_{26}H_{38}O_5Na[M + Na]^+$: 453.2614; found: 453.2614.

Pure yield of **32c**: 63mg, 94%; Yellow solid; M.P. 173-175 °C

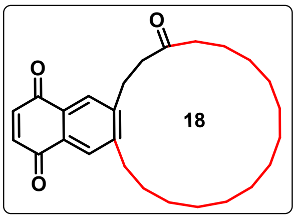

^{1}H NMR (400 MHz, $CDCl_3$): δ_H 7.85 (d, *J*=15.39 Hz, 2H), 6.91 (s, 2H), 3.03-2.99 (m, 2H), 2.75-2.70 (m, 4H), 2.46 (t, *J*=6.56 Hz, 2H), 1.67-1.59 (m, 6H), 1.44-1.25 (m, 16H); **^{13}C NMR** (100 MHz, $CDCl_3$):

δ_C 209.5, 185.3, 185.2, 148.2, 146.1, 138.8, 130.3, 130.1, 127.6, 127.2, 43.7, 42.1, 33.0, 31.0, 29.0, 28.0, 27.9, 27.79, 27.73, 27.3, 27.1, 26.9, 23.4; **HRMS** (ESI) m/z calcd. for $C_{26}H_{34}O_3Na[M + Na]^+$: 417.2399; found: 417.2399.

Pure yield of **32d**: 69mg, 91%; Yellow solid; M.P. 155-157 °C

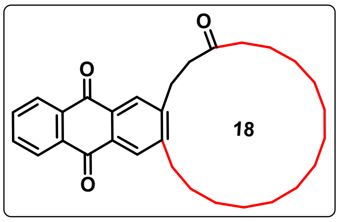

^{1}H NMR (500 MHz, $CDCl_3$): δ_H 8.26-8.24 (m, 2H), 8.03 (d, *J*=17.94 Hz, 2H), 7.76-7.74 (m, 2H), 3.03-3.00 (m, 2H), 2.77-2.71 (m, 4H), 2.46 (t, *J*=6.62 Hz, 2H), 1.66-1.60 (m, 4H), 1.44-1.26 (m, 18H); **^{13}C NMR** (125 MHz, $CDCl_3$): δ_C 209.6, 183.27, 183.20, 148.4, 146.3, 134.0, 133.75, 133.73, 131.8, 131.5, 128.3, 127.9, 127.2, 43.8, 42.0, 32.9, 31.0, 29.0, 27.96, 27.91, 27.7, 27.6, 27.2, 27.1, 27.0, 26.86, 26.83, 23.4; **HRMS** (ESI) m/z calcd. for $C_{30}H_{36}O_3Na[M + Na]^+$: 467.2561; found: 467.2560.

[1]**H NMR** (500 MHz, $CDCl_3$) of Compound **14**

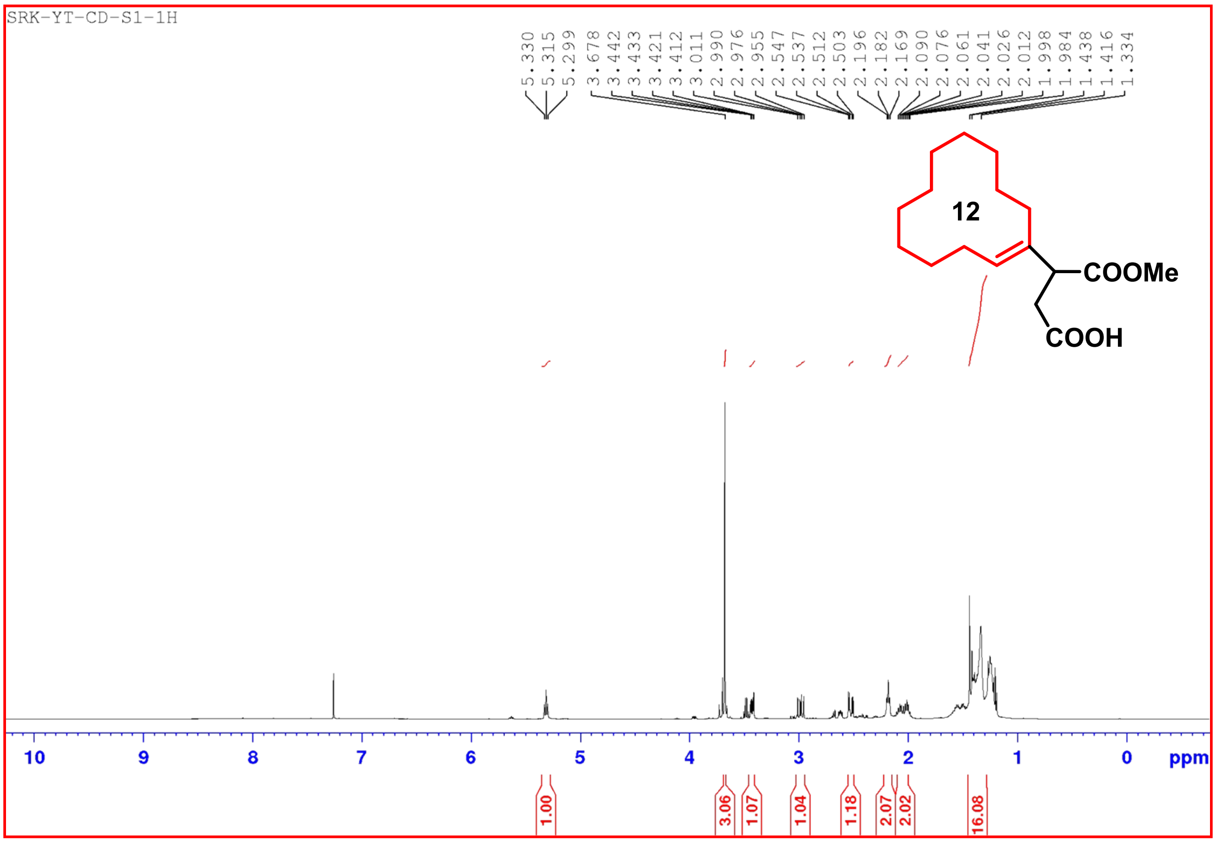

[13]**C NMR** (125 MHz, $CDCl_3$) of compound **14**

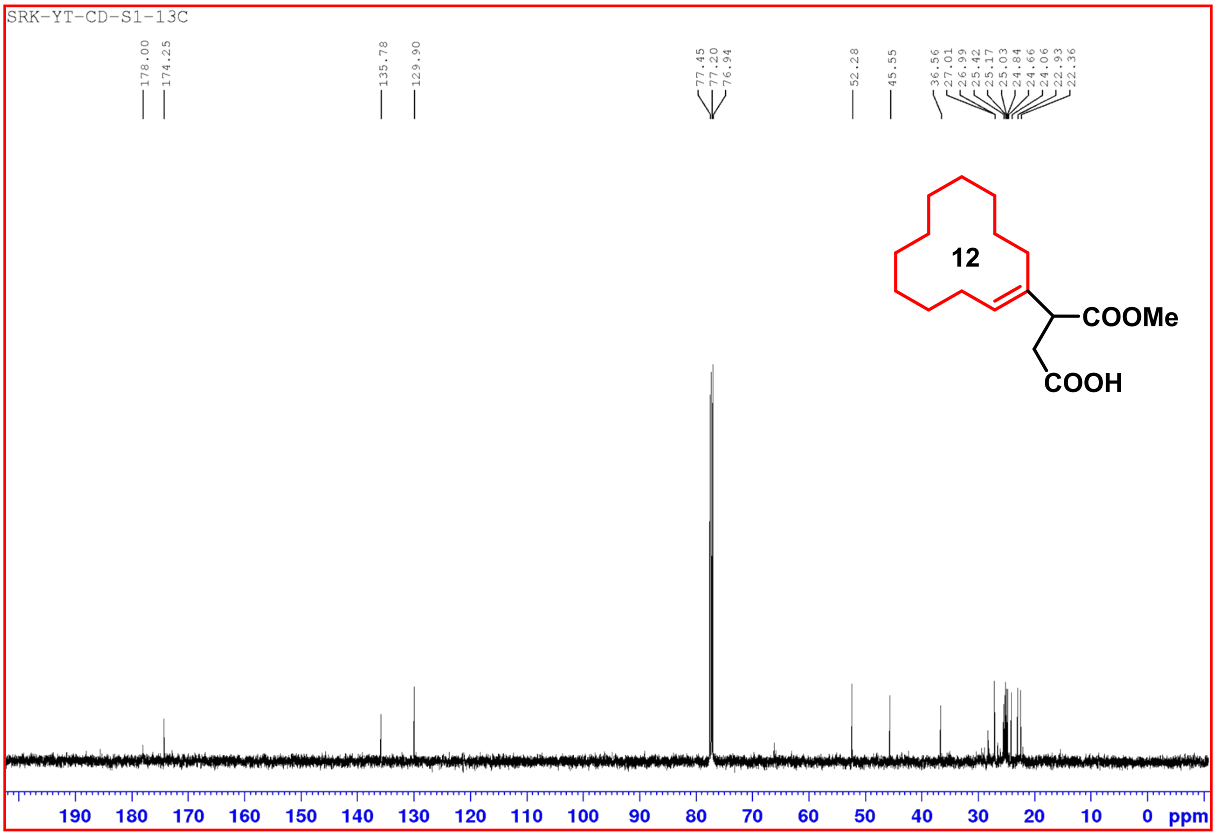

^{1}H NMR (500 MHz, $CDCl_3$) of compound **15**

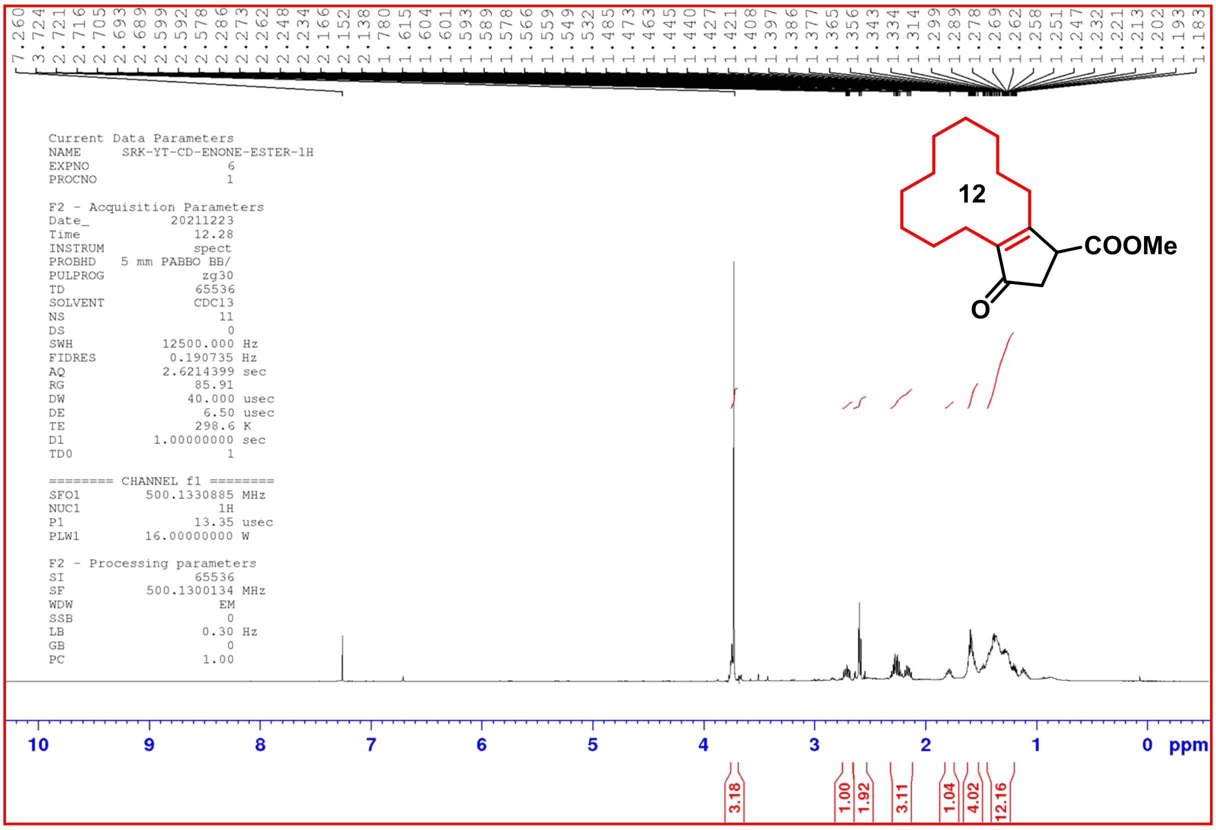

^{13}C NMR (125 MHz, $CDCl_3$) of compound **15**

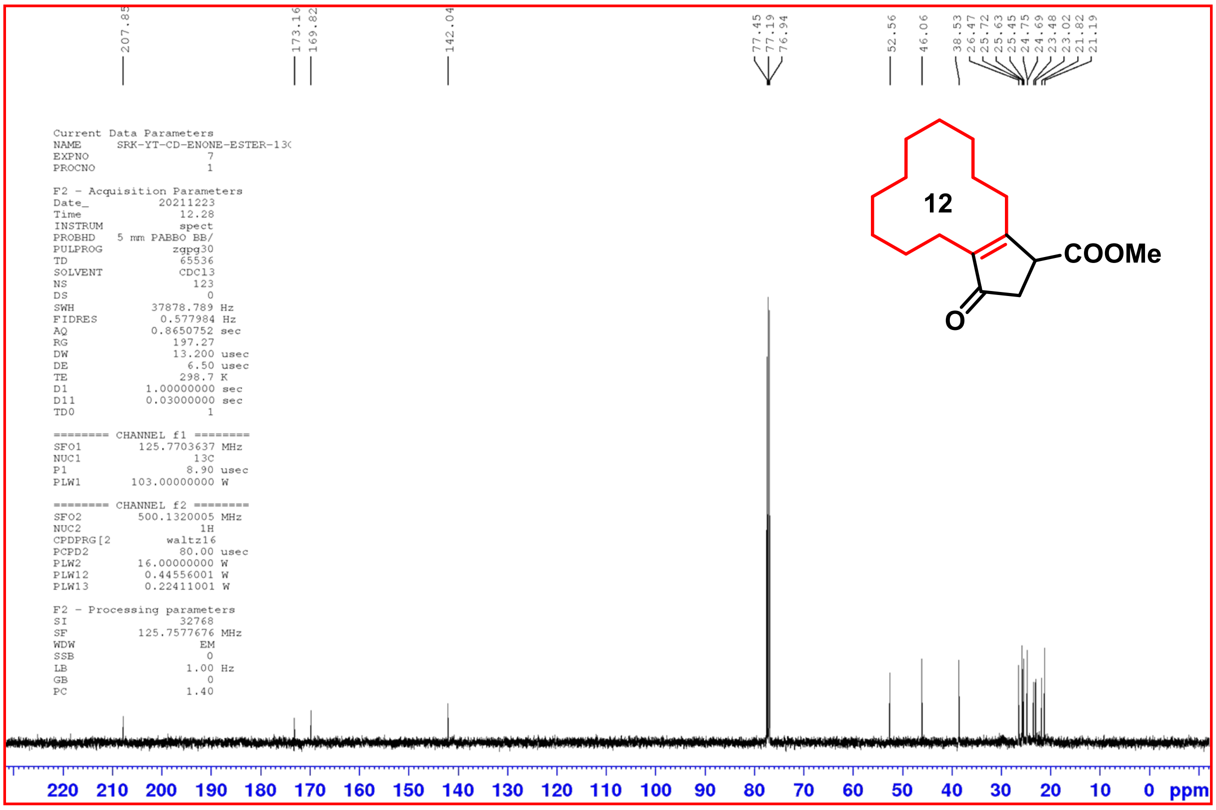

^{1}H NMR (500 MHz, $CDCl_3$) of compound **16**

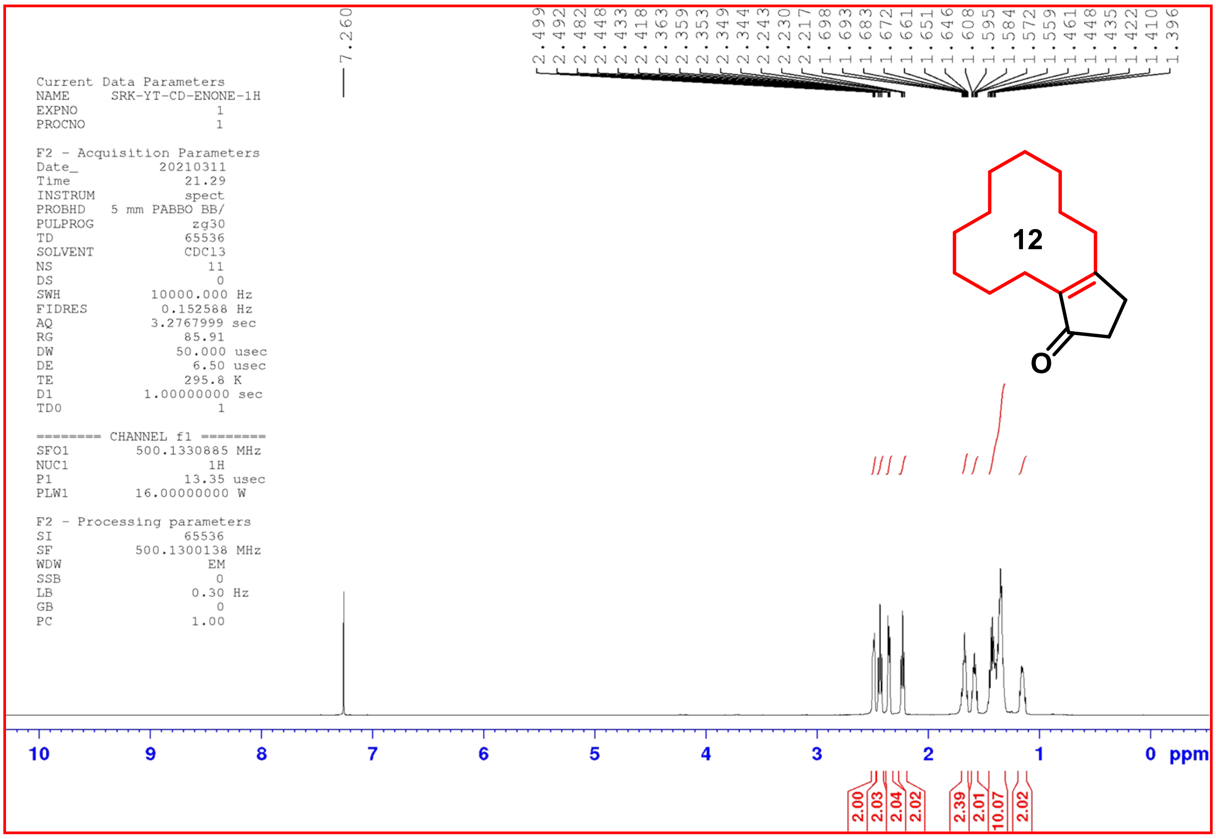

^{13}C NMR (125 MHz, $CDCl_3$) 12 of compound **16**

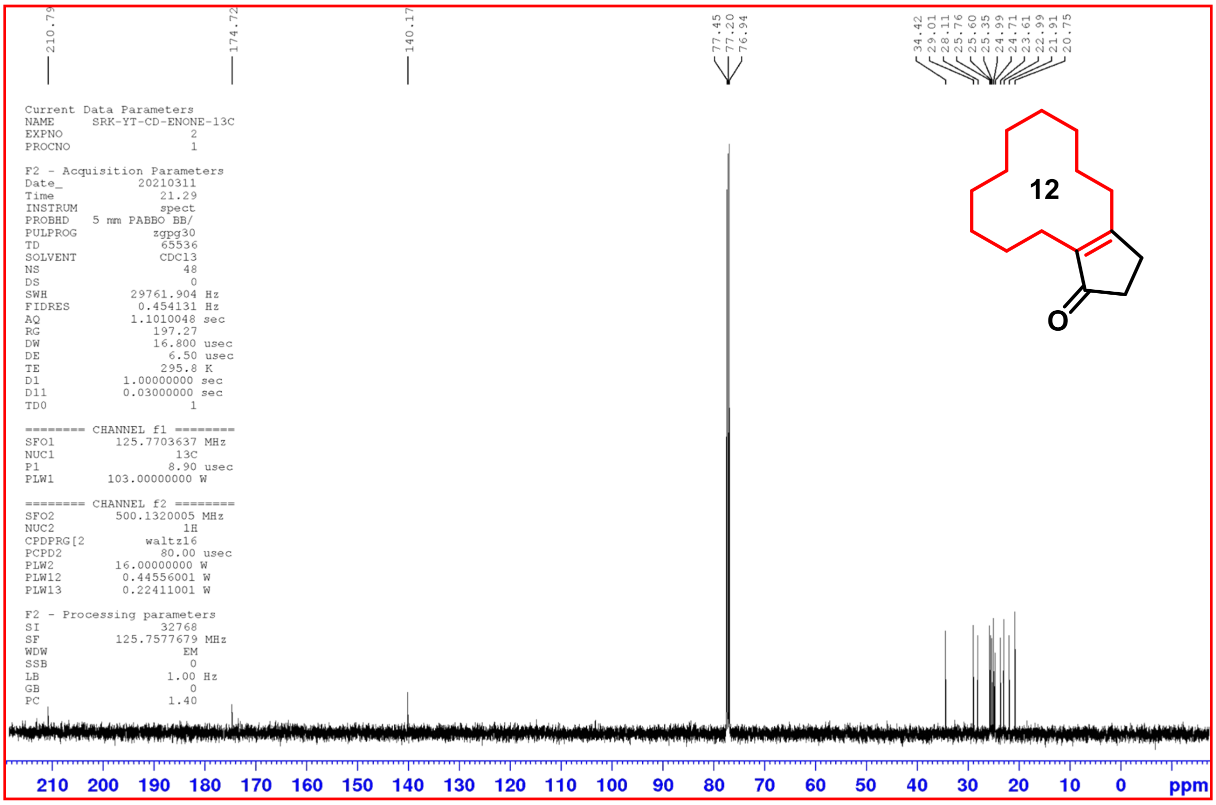

^{1}H NMR (500 MHz, $CDCl_3$) of compound **18**

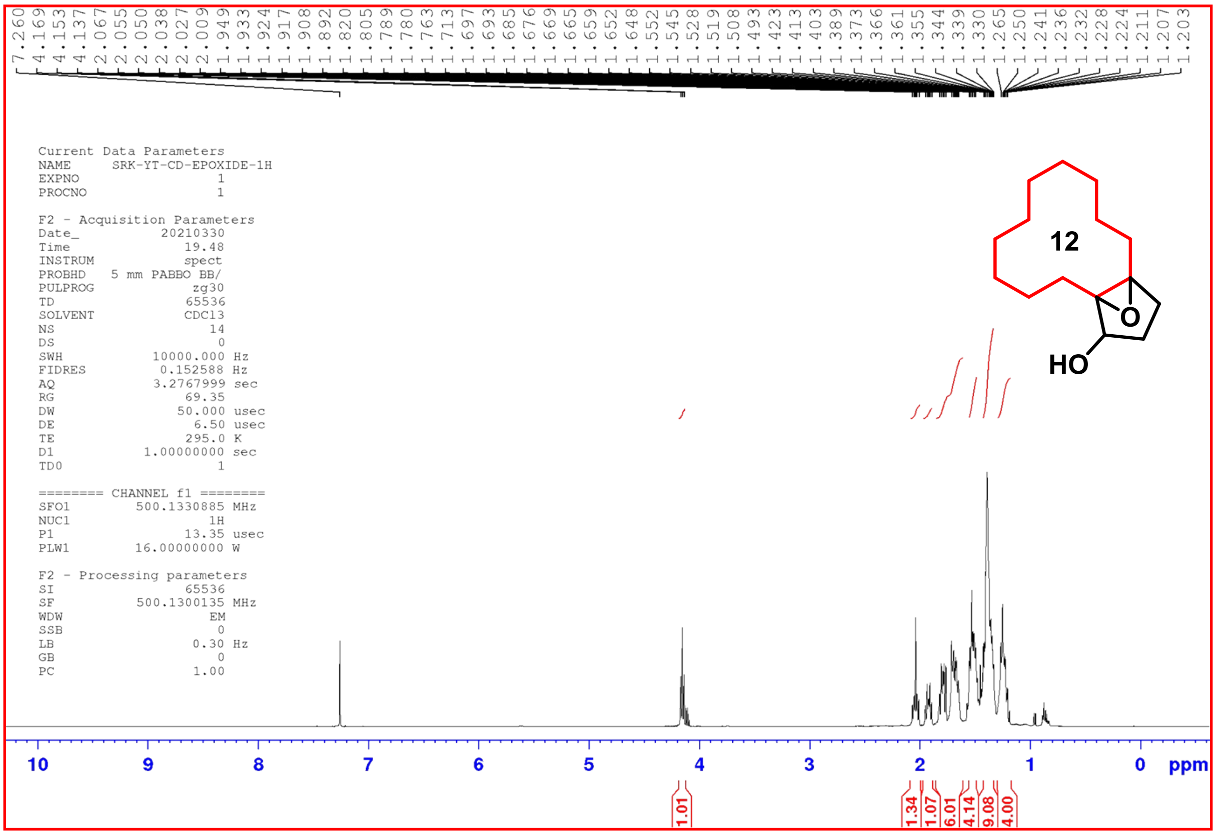

^{13}C NMR (125 MHz, $CDCl_3$) of compound **18**

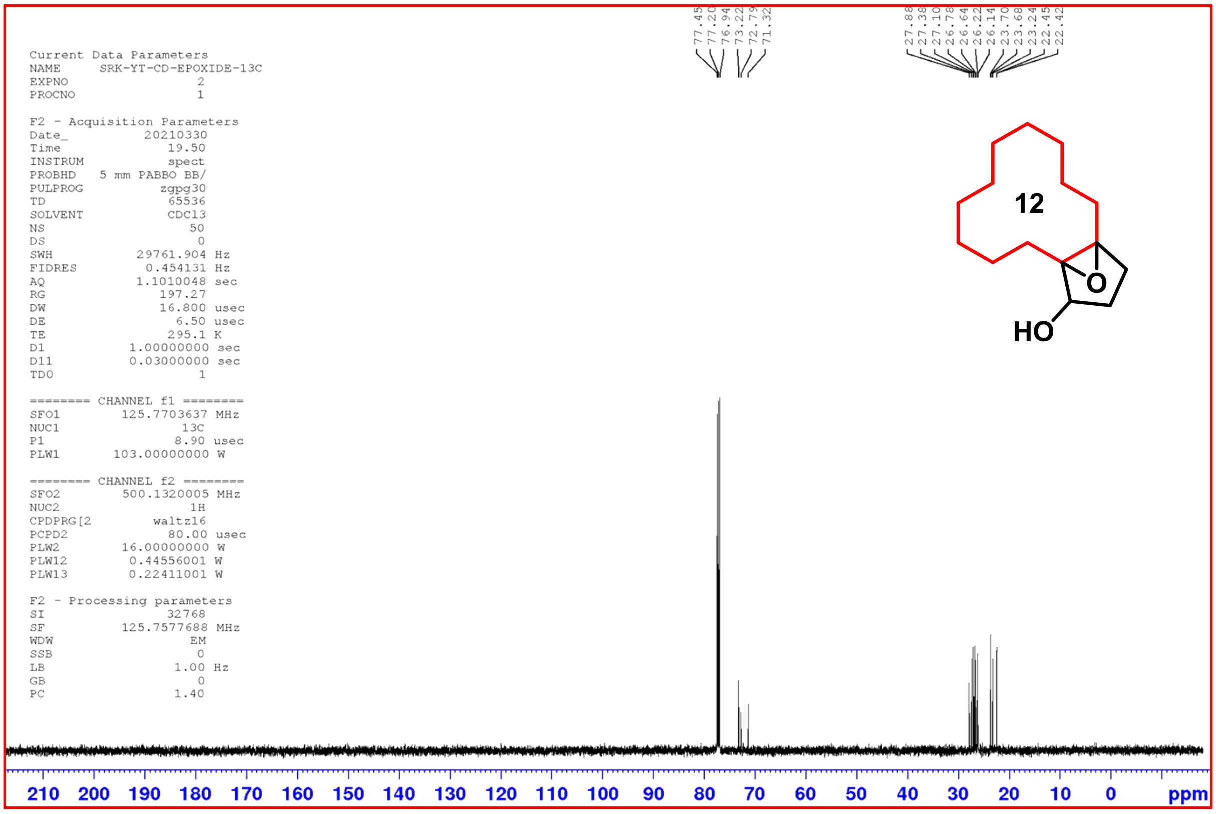

[1]**H NMR** (500 MHz, $CDCl_3$) of compound **19**

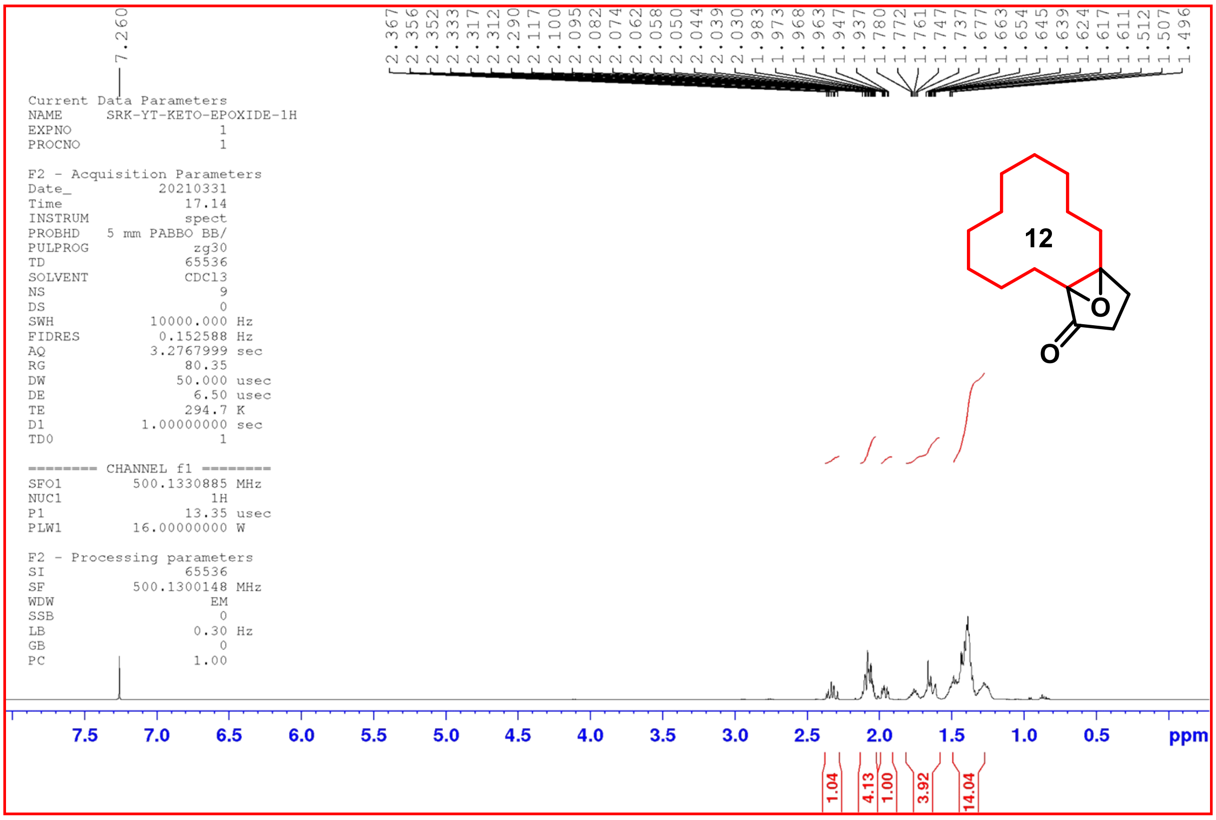

[13]**C NMR** (125 MHz, $CDCl_3$) of compound **19**

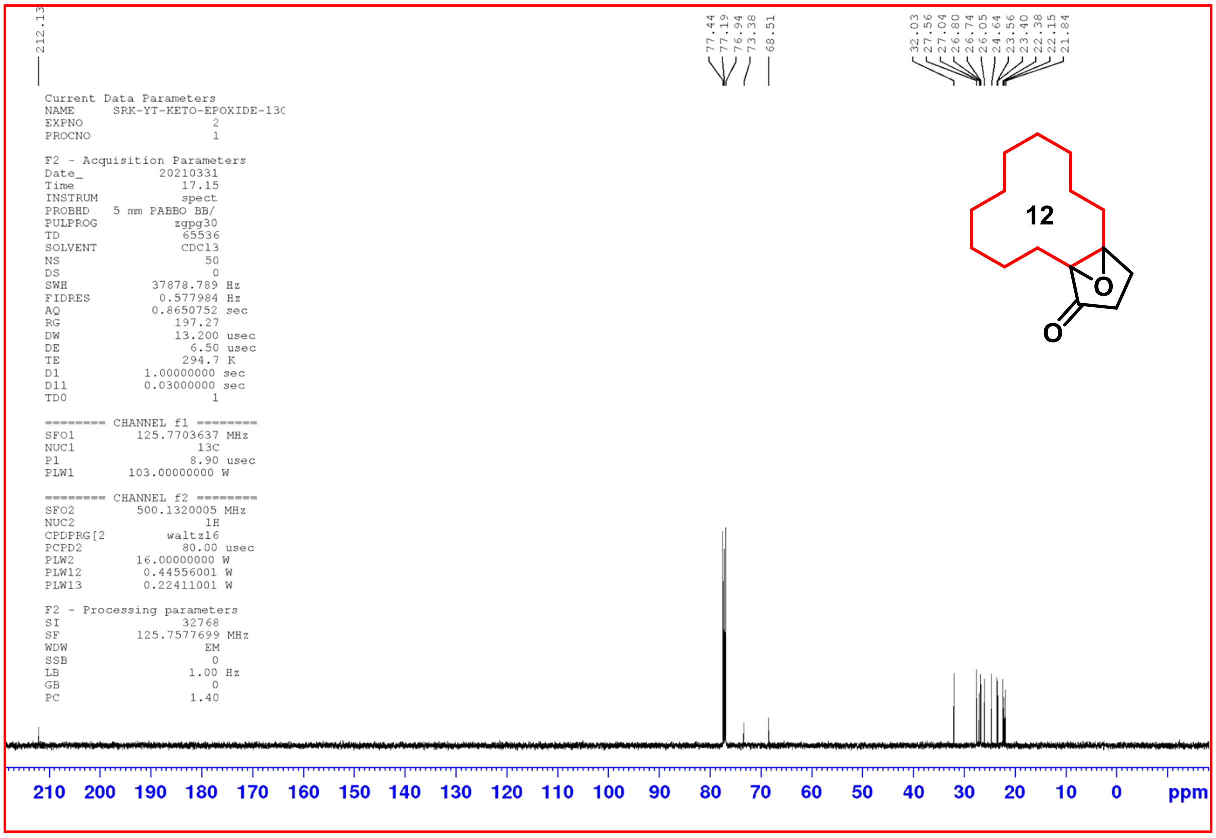

^{1}H **NMR** (500 MHz, $CDCl_3$) of compound **20**

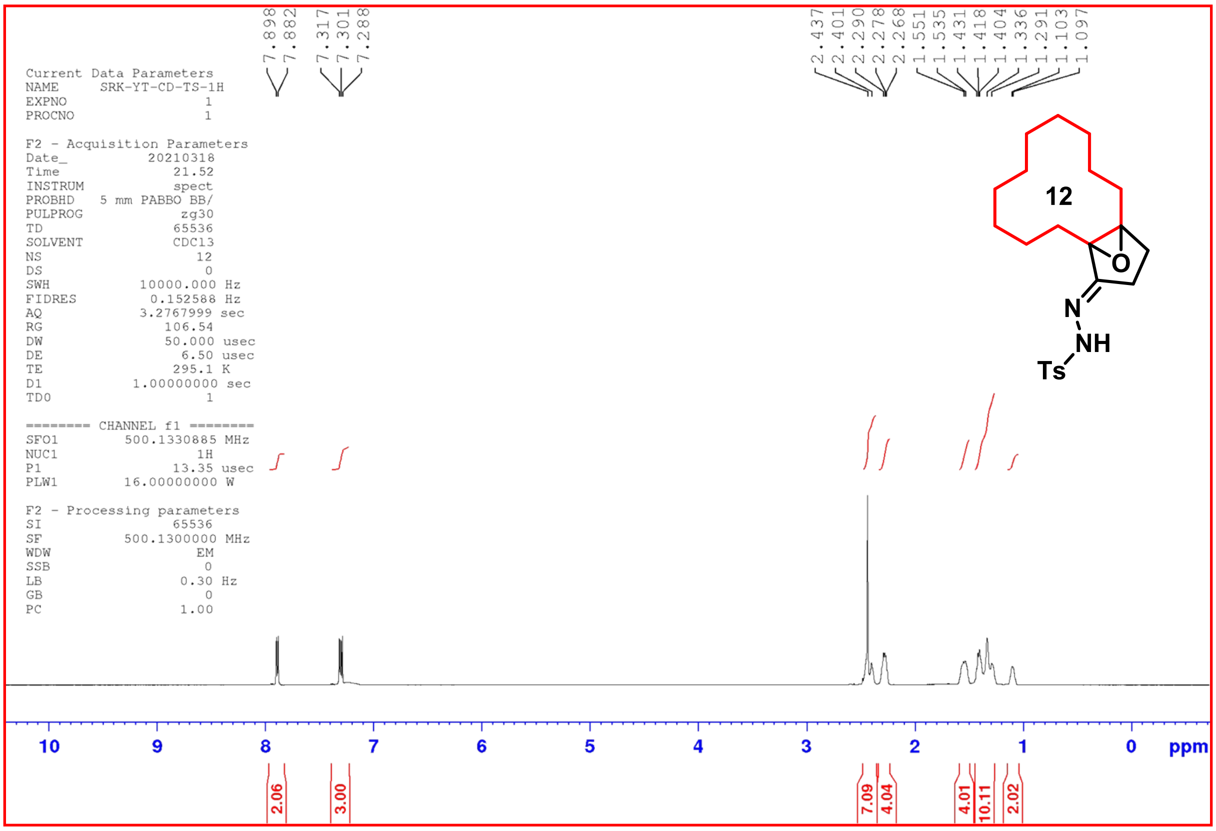

^{13}C **NMR** (125 MHz, $CDCl_3$) 12 of compound **20**

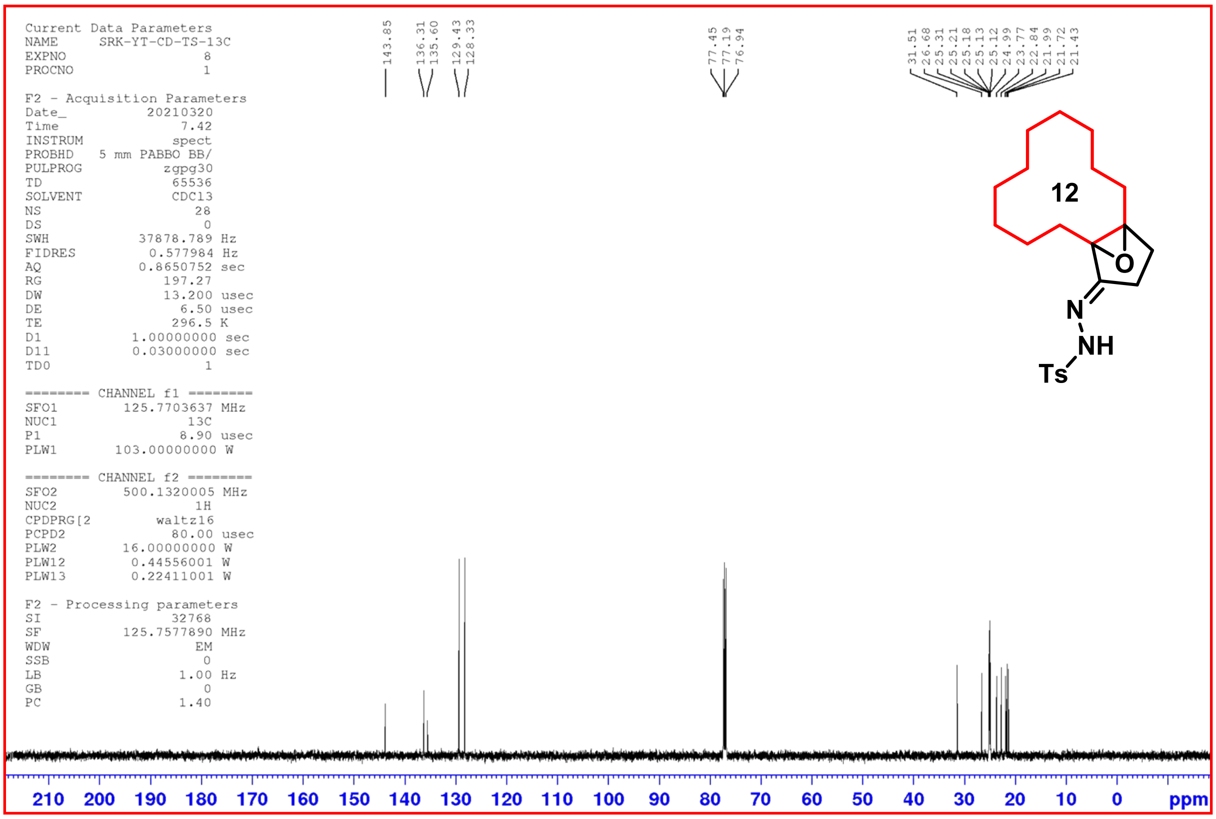

^{1}H NMR (500 MHz, $CDCl_3$) of compound **21**

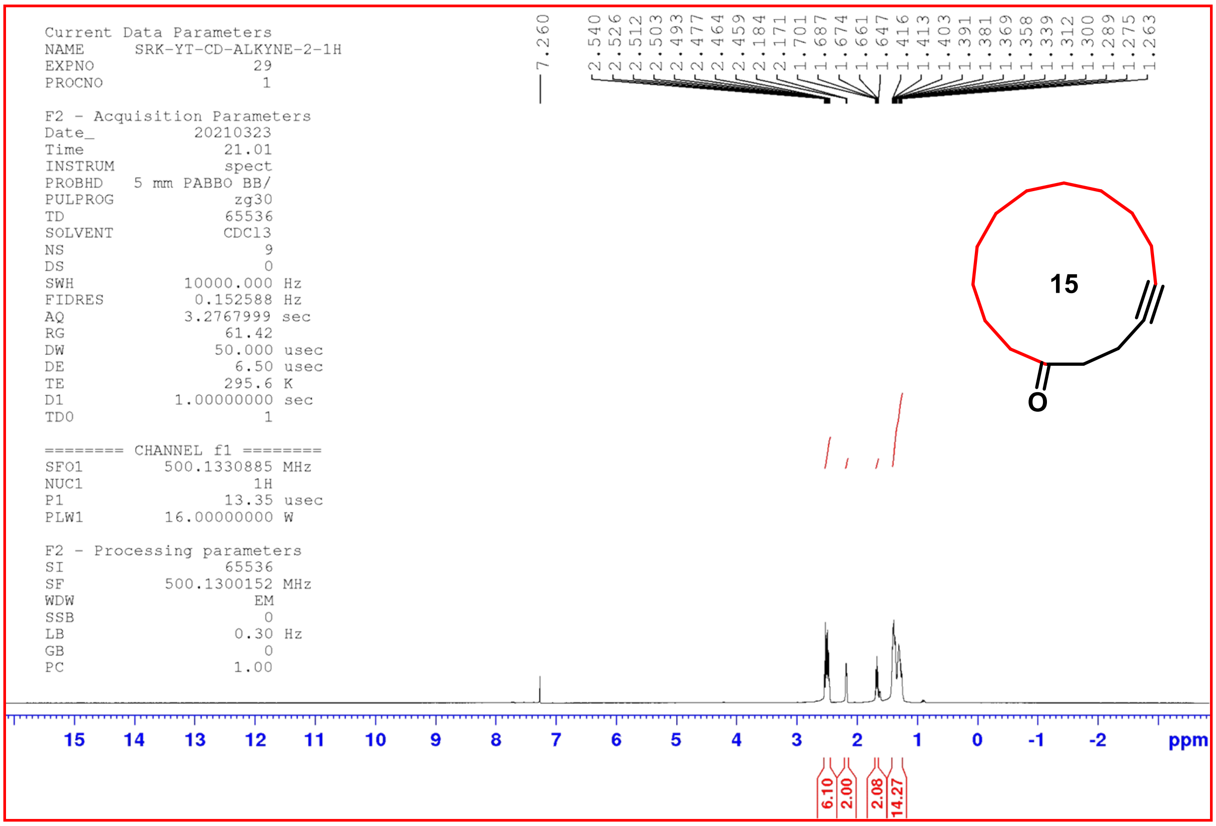

^{13}C NMR (125 MHz, $CDCl_3$) of compound **21**

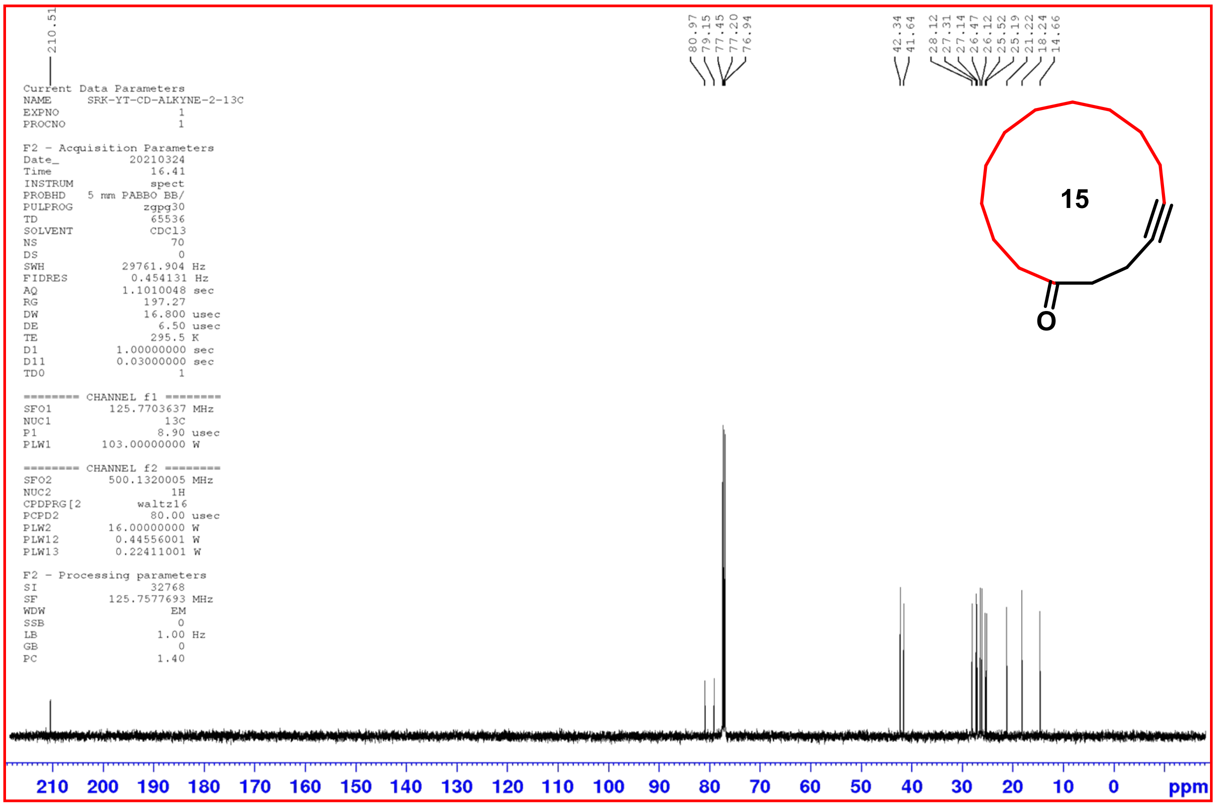

^{1}H NMR (400 MHz, $CDCl_3$) of compound **22**

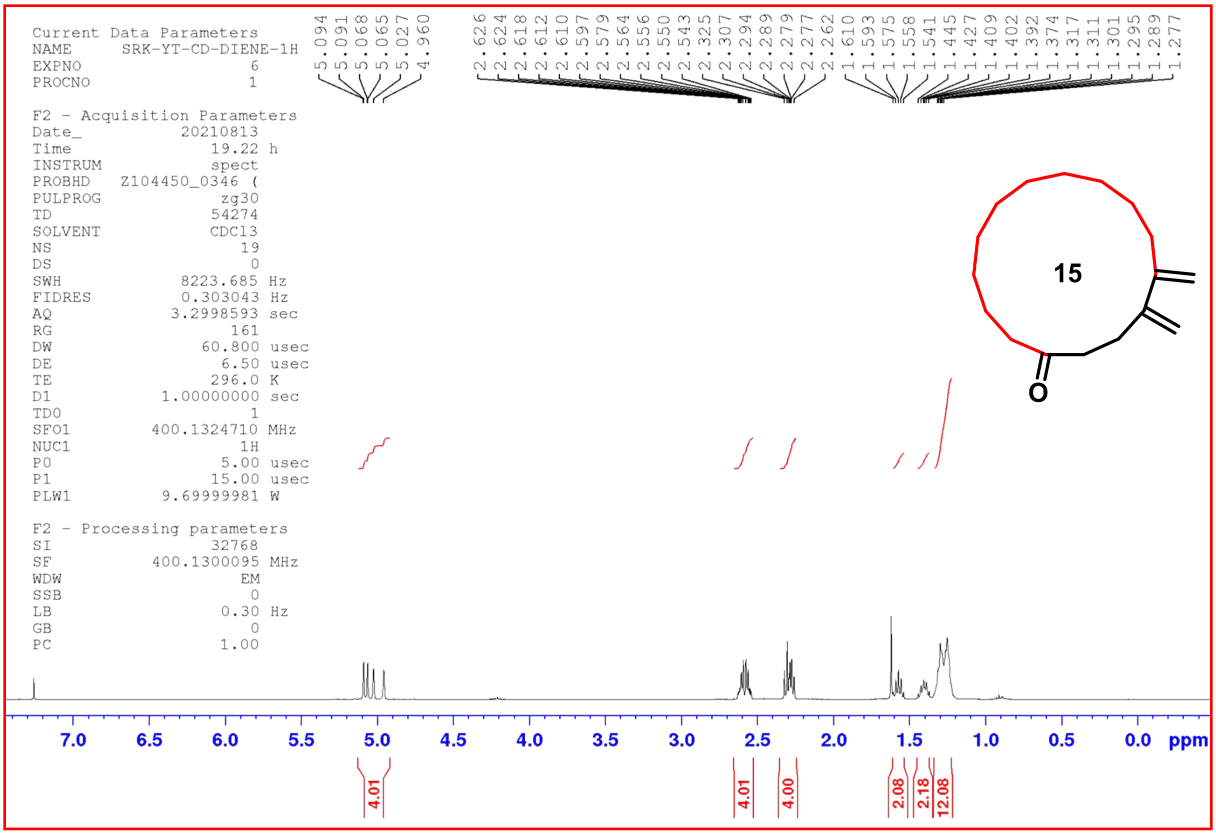

^{13}**C NMR** (100 MHz, $CDCl_3$) of compound **22**

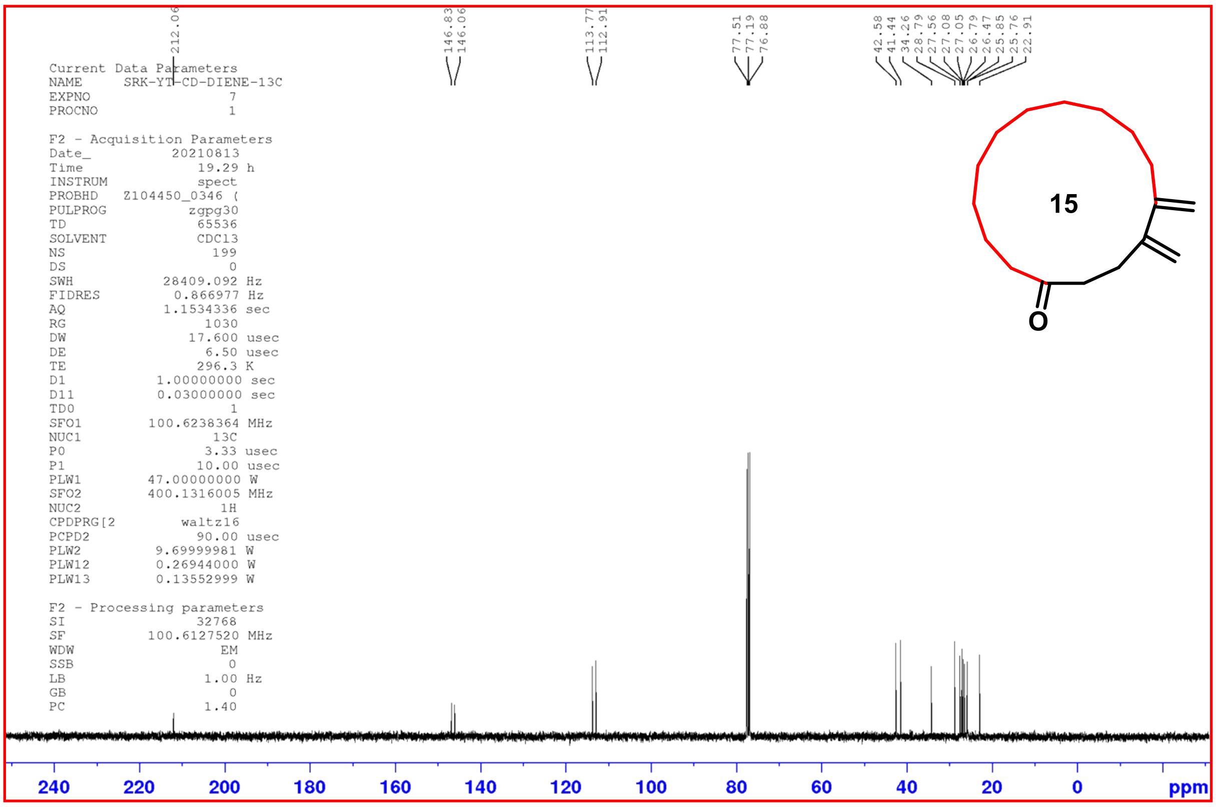

^{1}**H NMR** (400 MHz, $CDCl_3$) of compound **23a**

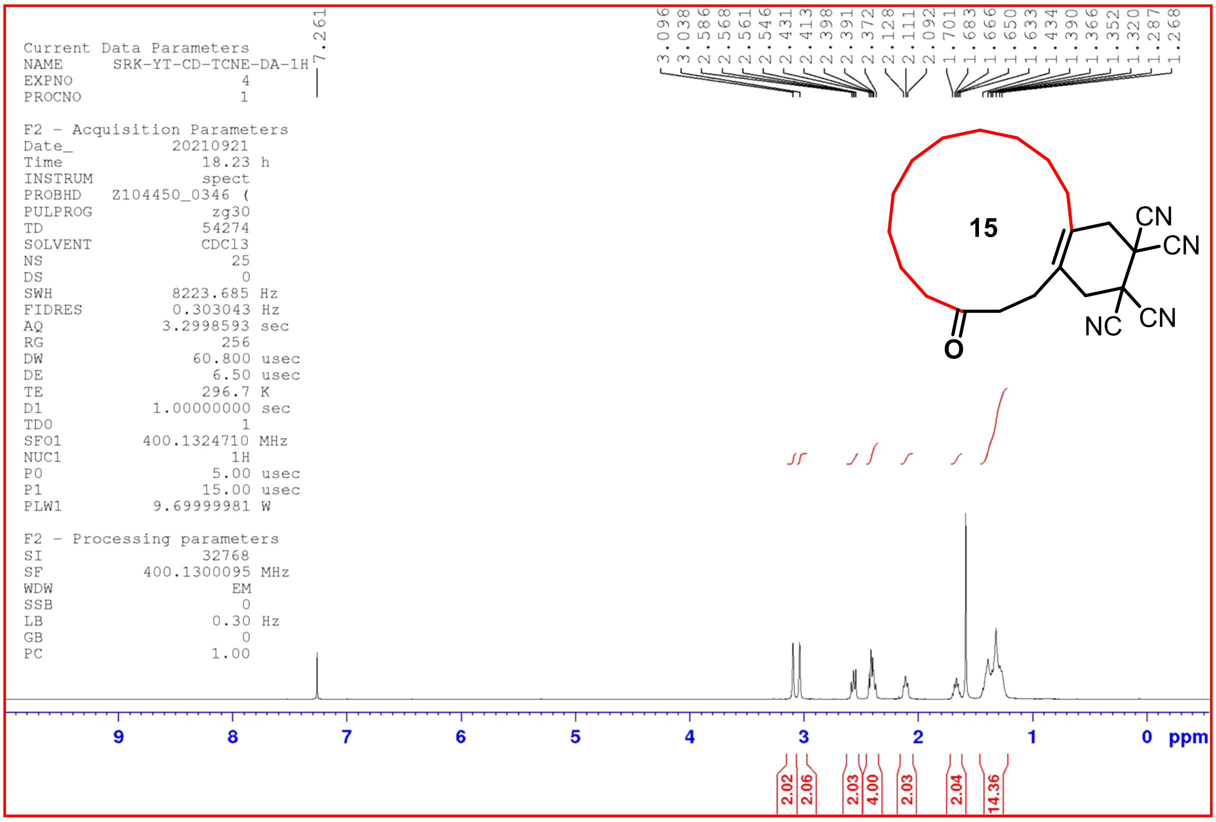

^{13}C NMR (100 MHz, $CDCl_3$) of compound **23a**

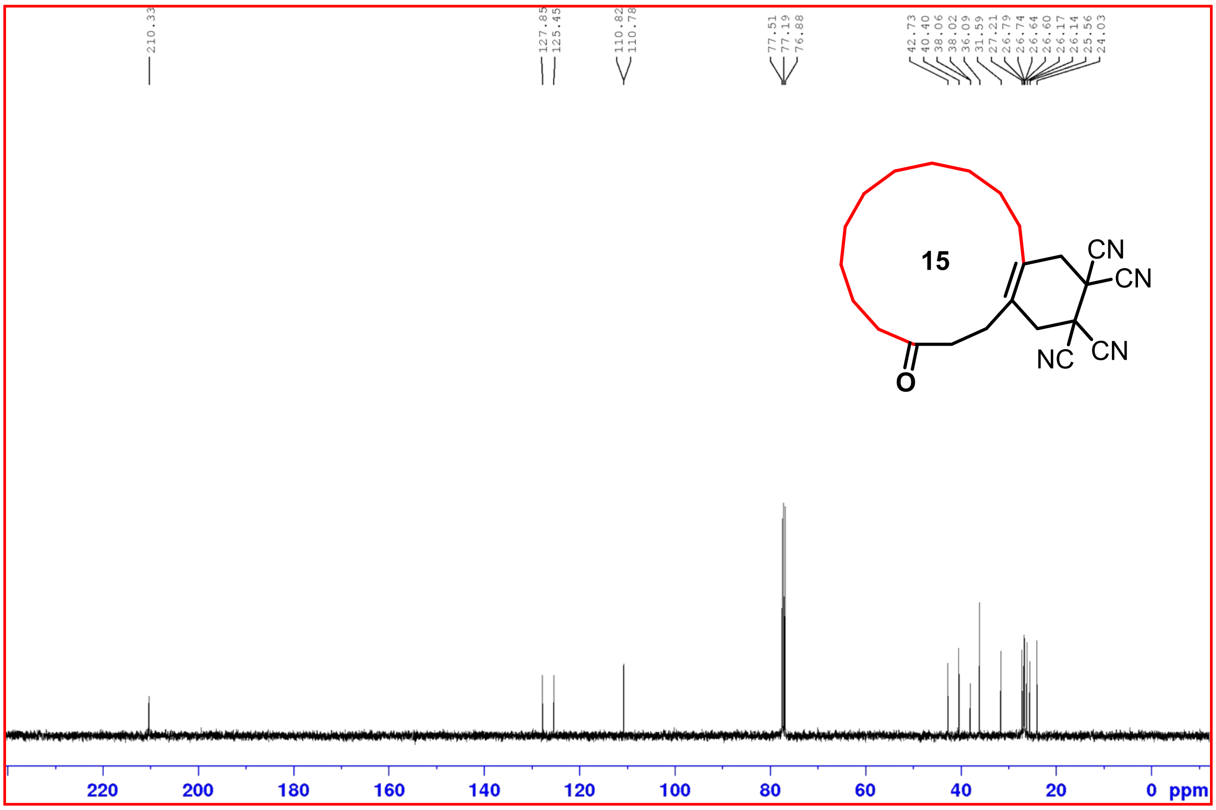

^{1}H NMR (400 MHz, $CDCl_3$) of compound **23b**

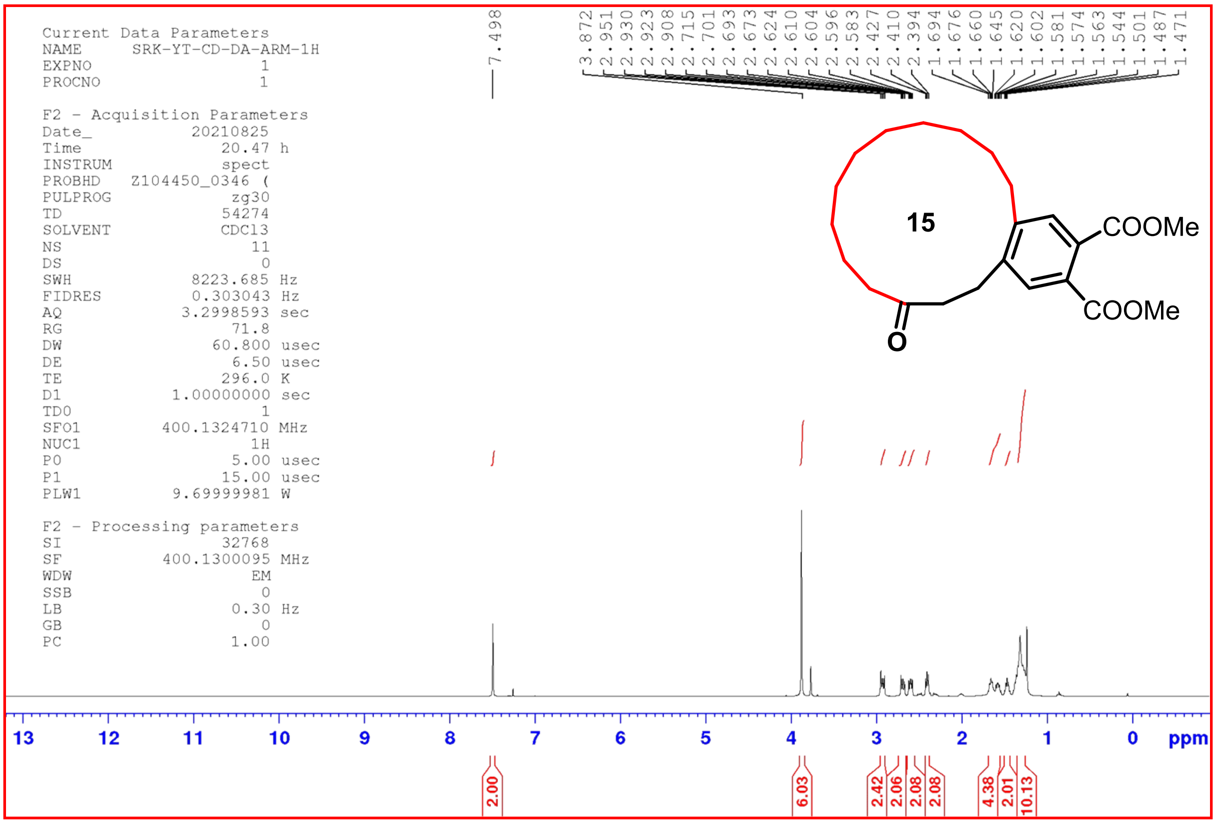

^{13}C NMR (100 MHz, $CDCl_3$) of compound **23b**

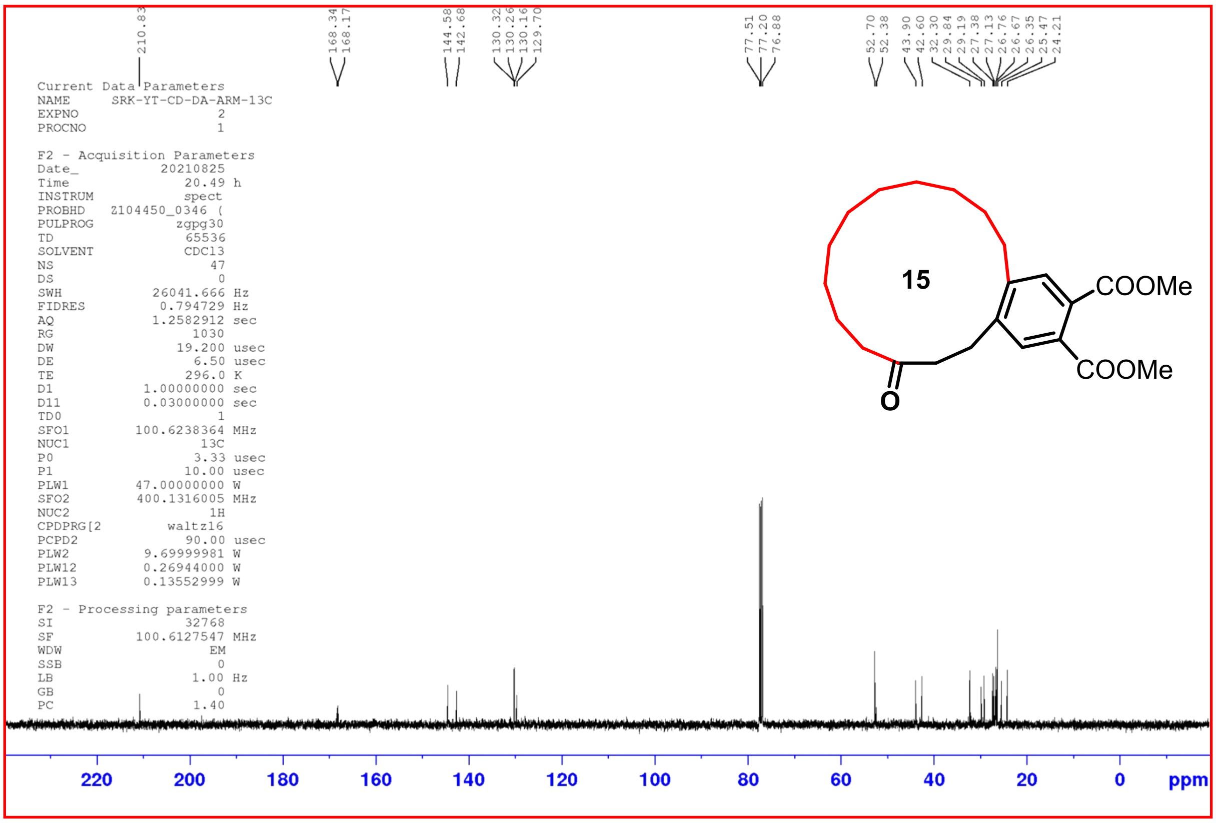

^{1}H NMR (400 MHz, $CDCl_3$) of compound **23c**

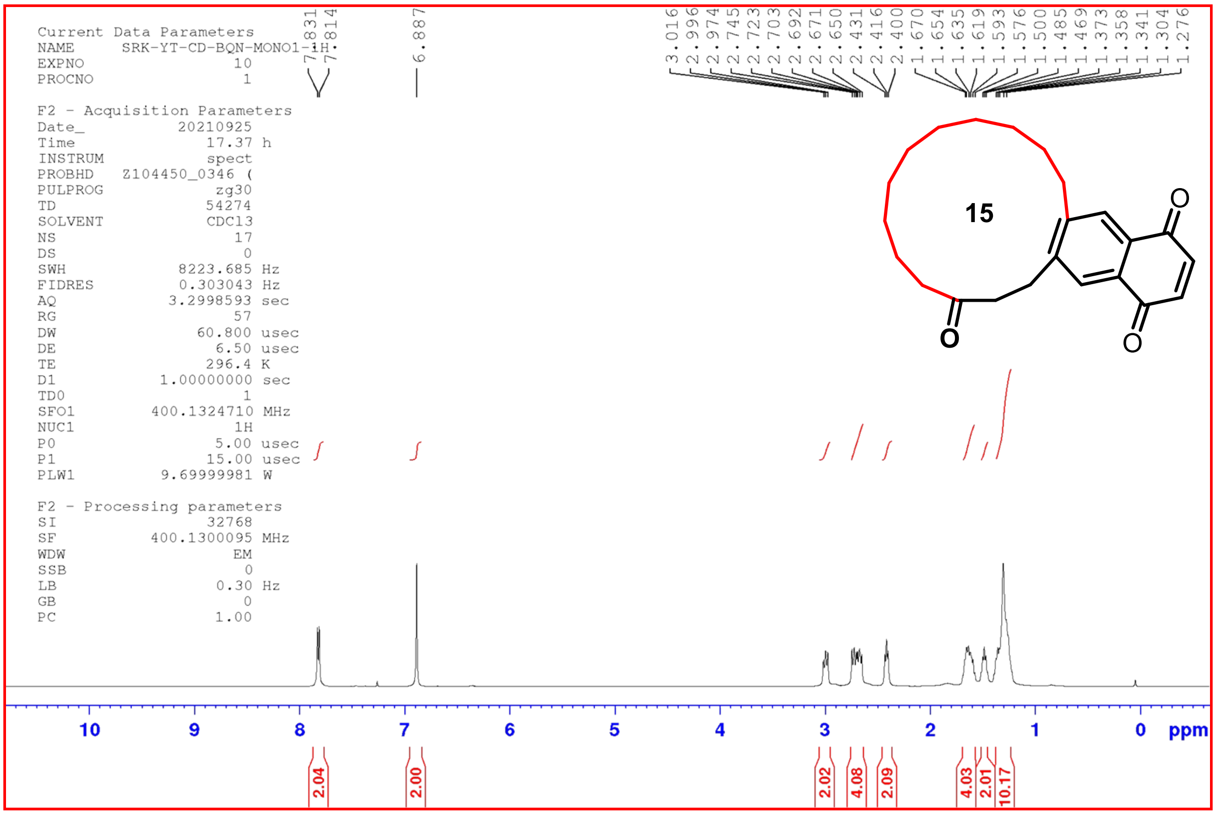

^{13}C NMR (100 MHz, $CDCl_3$) of compound **23c**

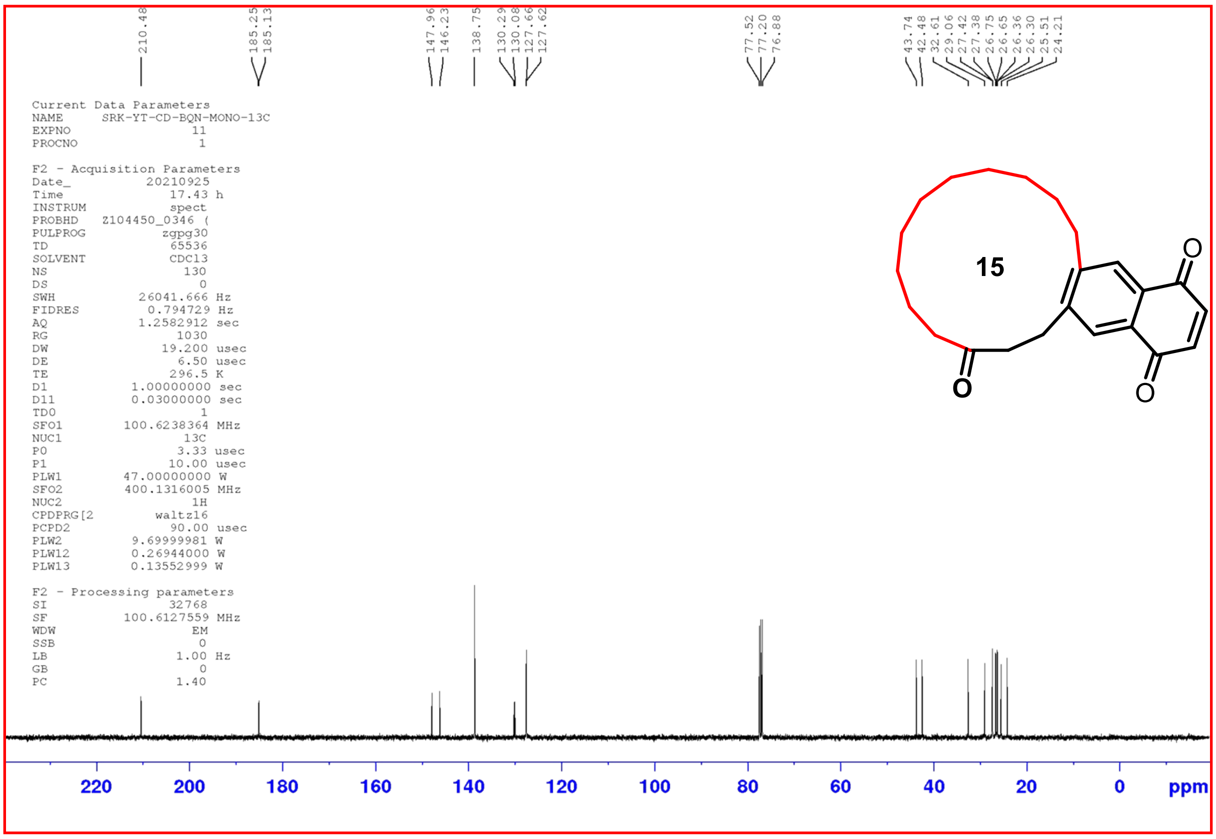

^{1}H NMR (500 MHz, $CDCl_3$) of compound **23d**

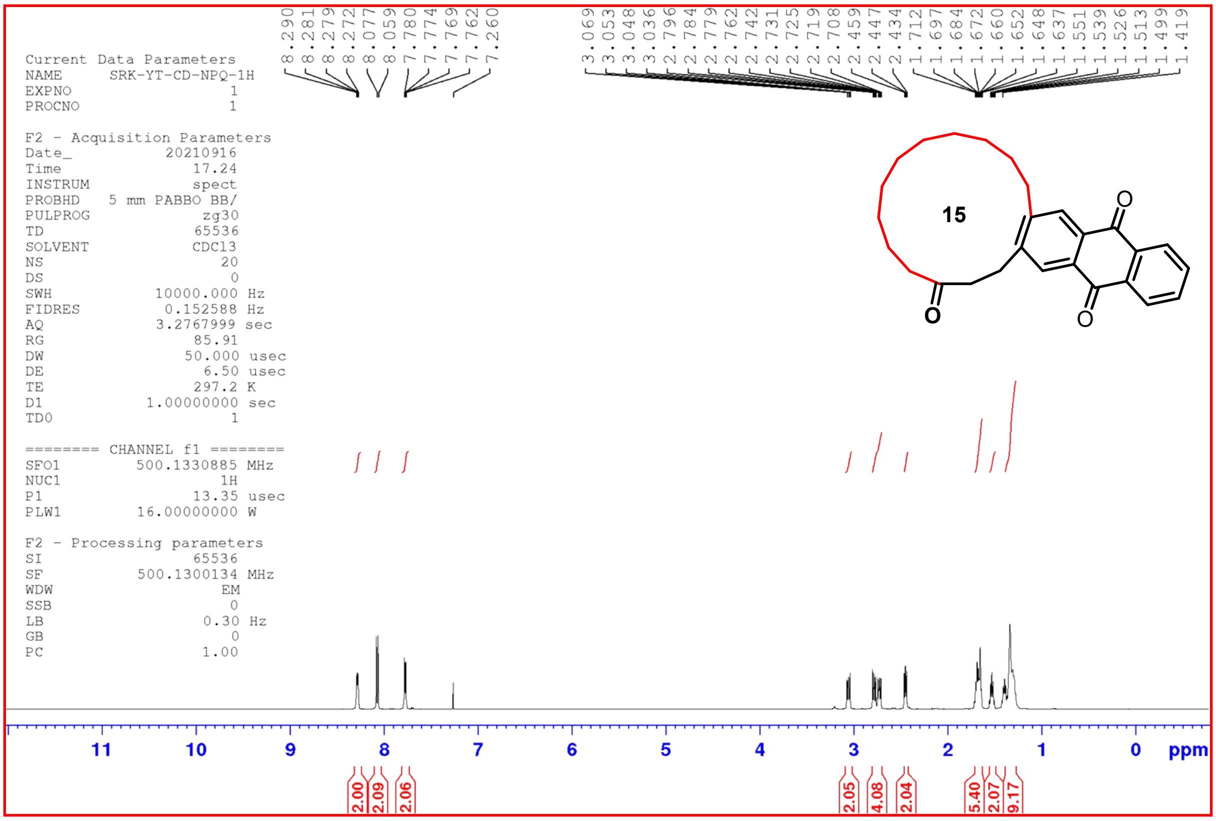

^{13}C NMR (125 MHz, CDCl$_3$) of compound **23d**

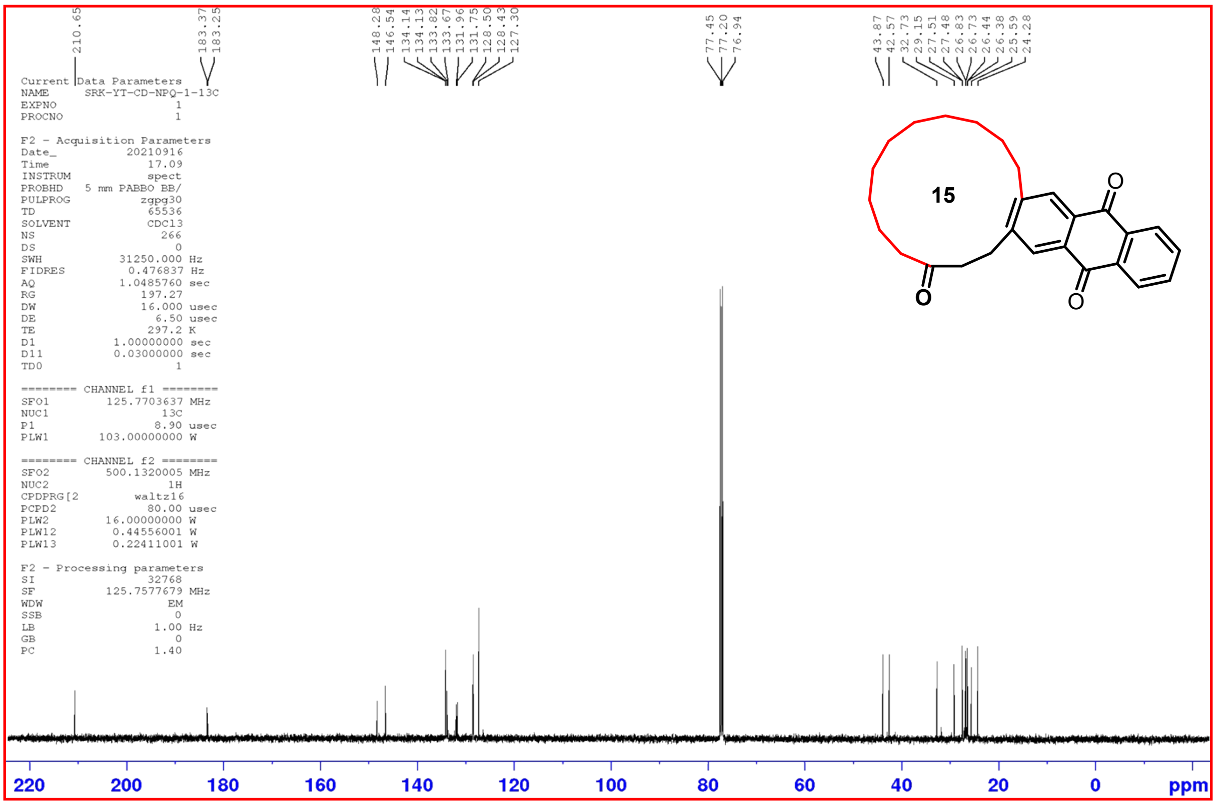

^{1}H NMR (400 MHz, CDCl$_3$) of compound **25**

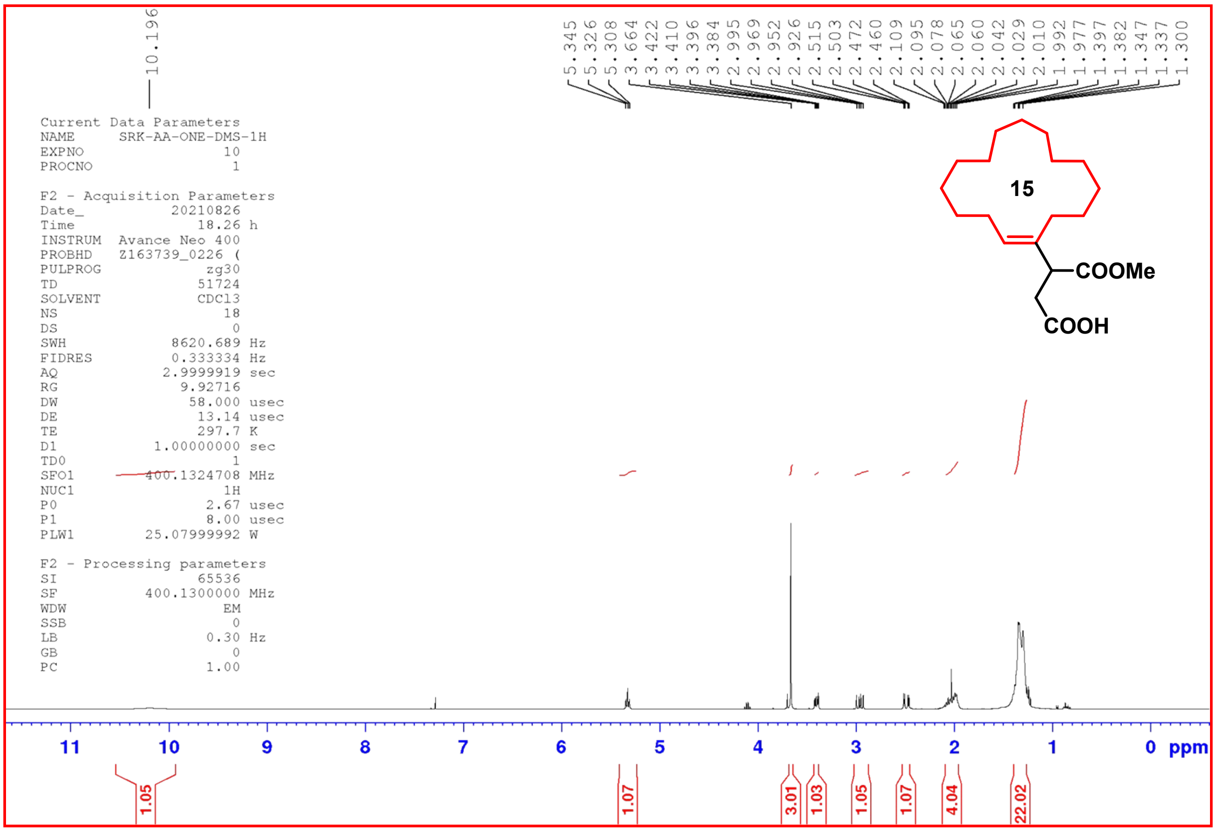

^{13}C NMR (100 MHz, $CDCl_3$) of compound **25**

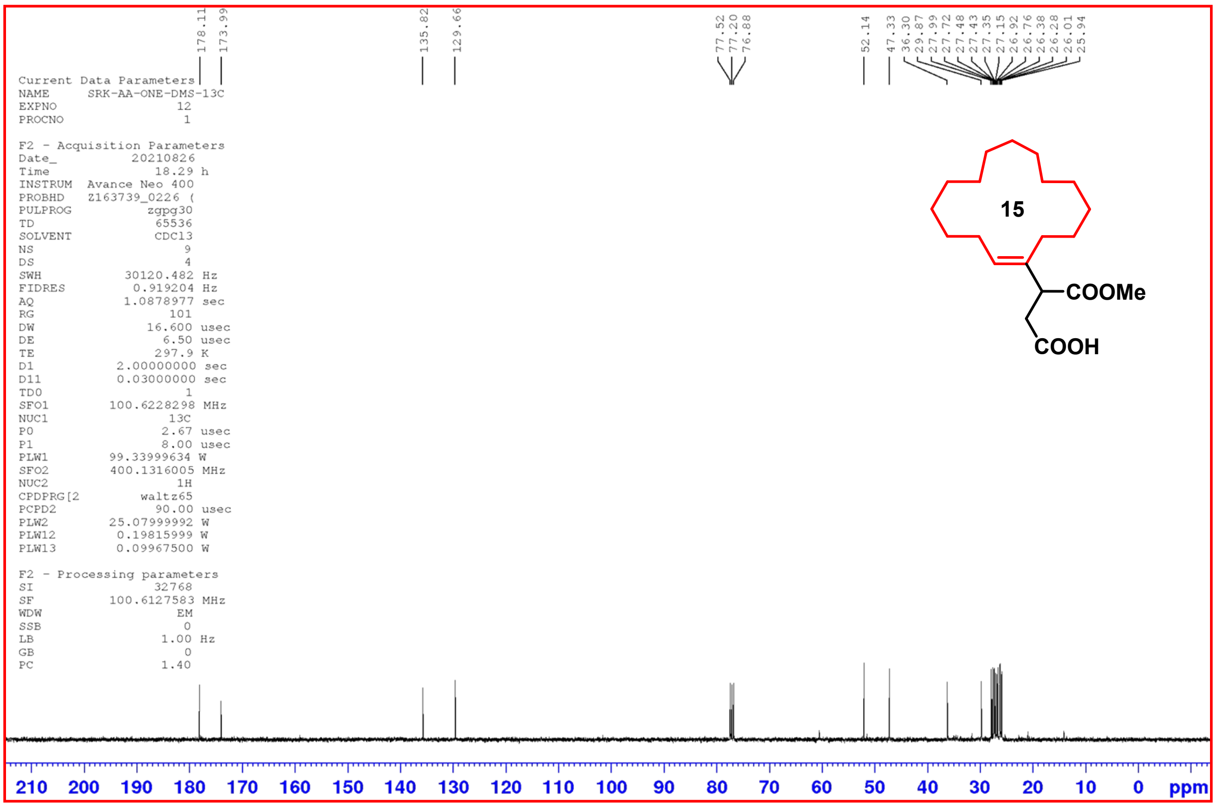

^{1}H NMR (400 MHz, $CDCl_3$) of compound **26**

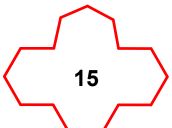

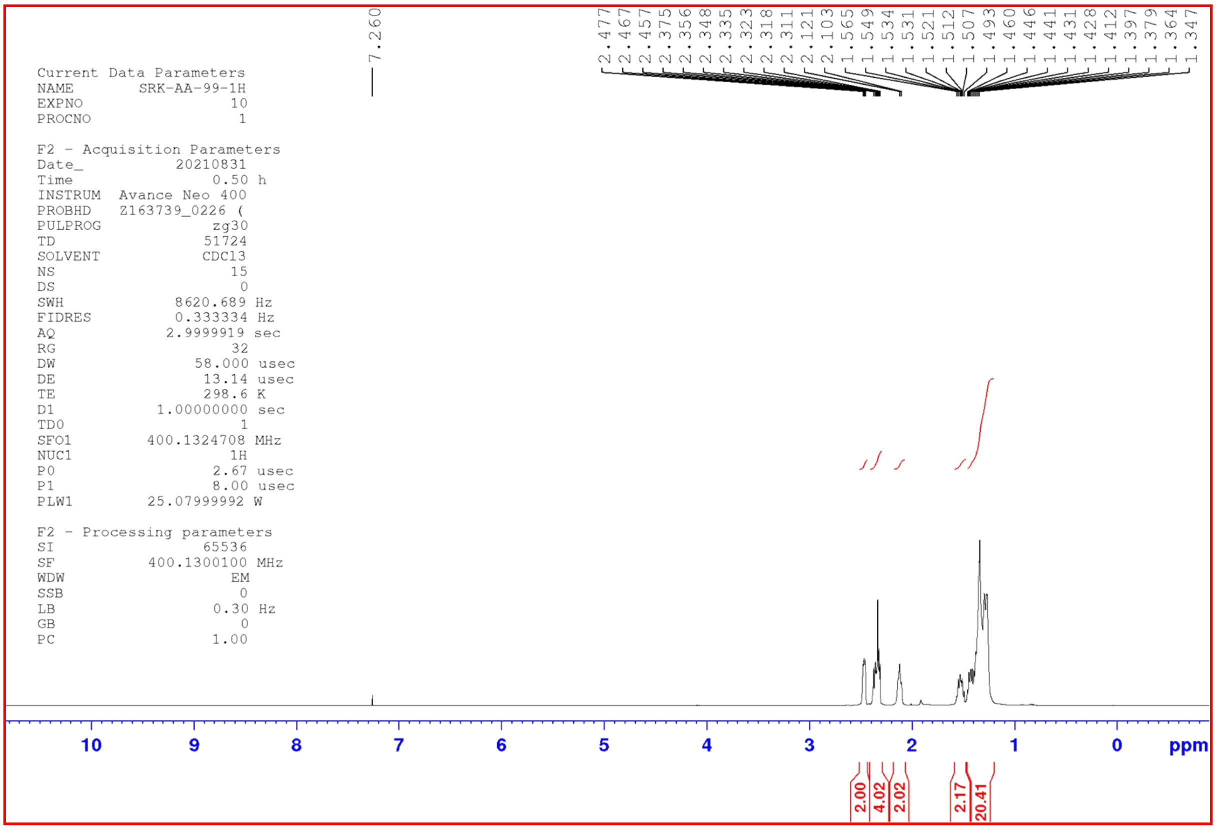

^{13}**C NMR** (100 MHz, $CDCl_3$) of compound **26**

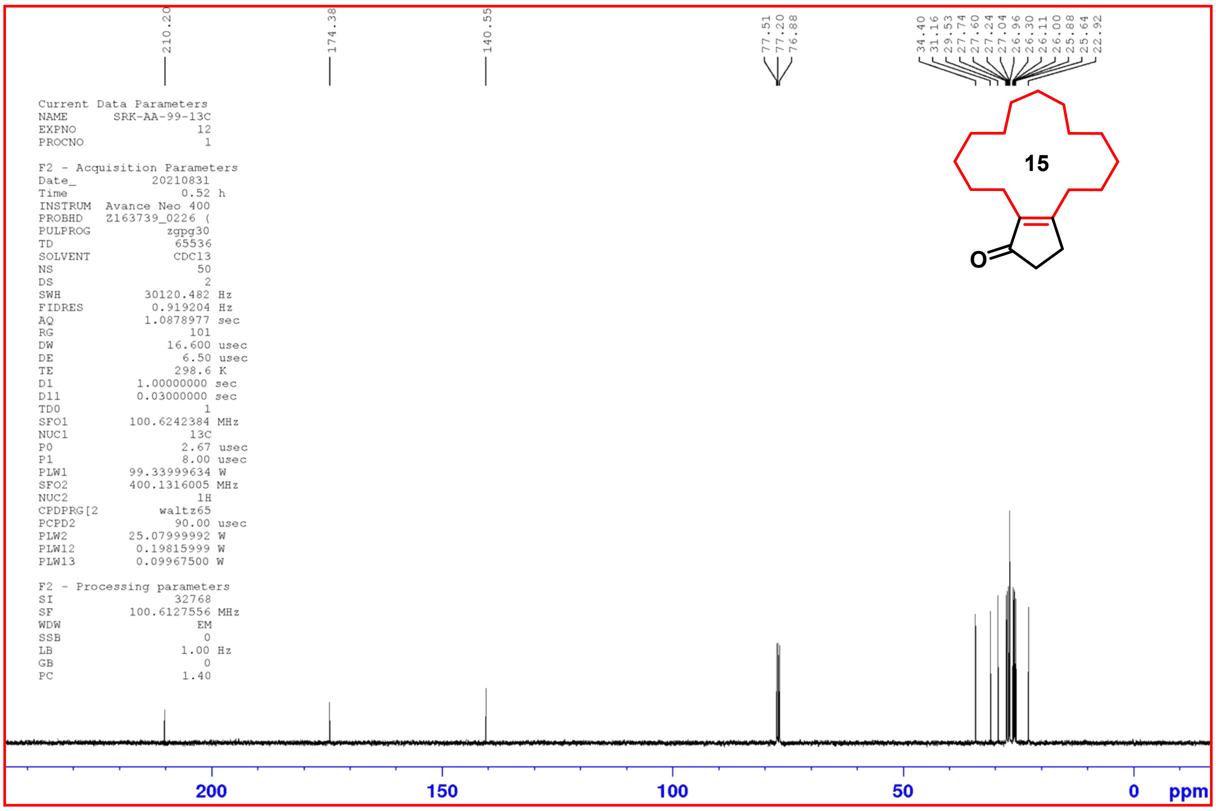

^{1}**H NMR** (400 MHz, $CDCl_3$) of compound **28**

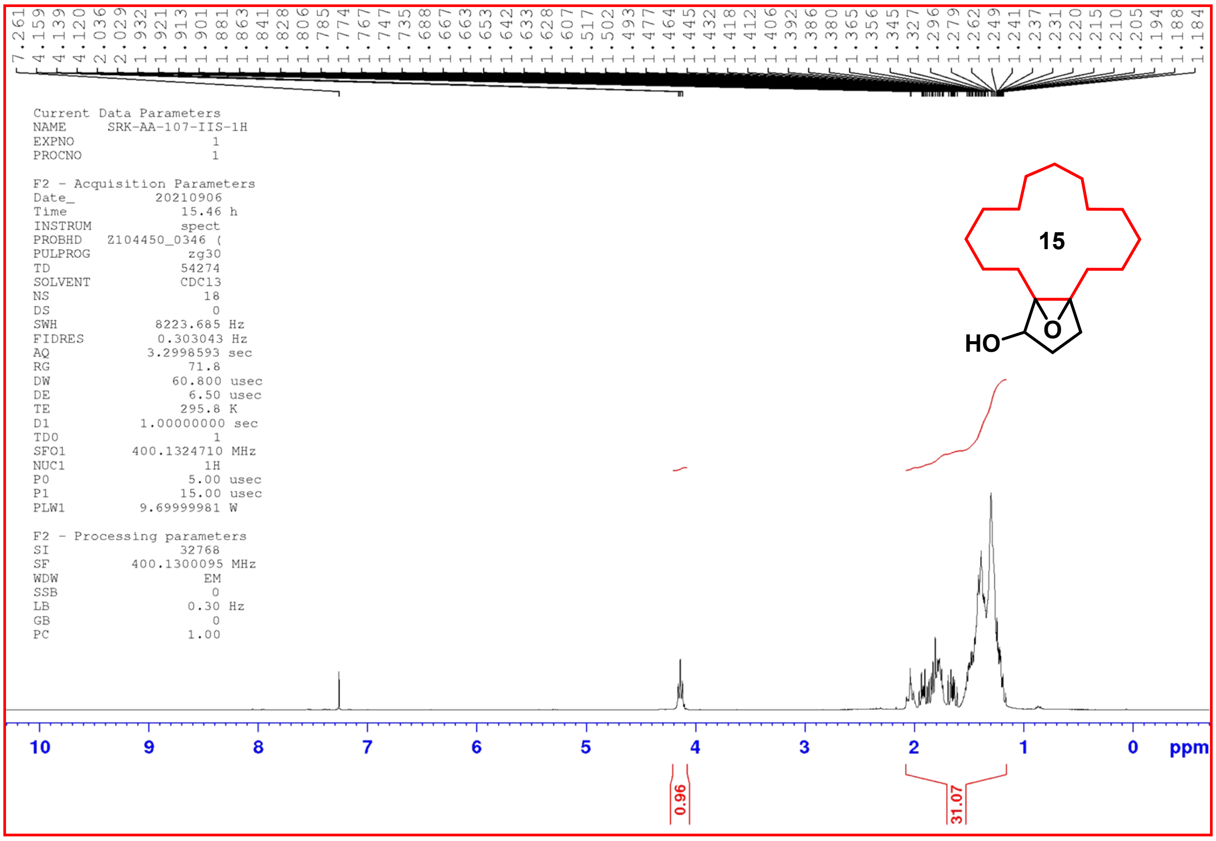

^{13}**C NMR** (100 MHz, $CDCl_3$) of compound **28**

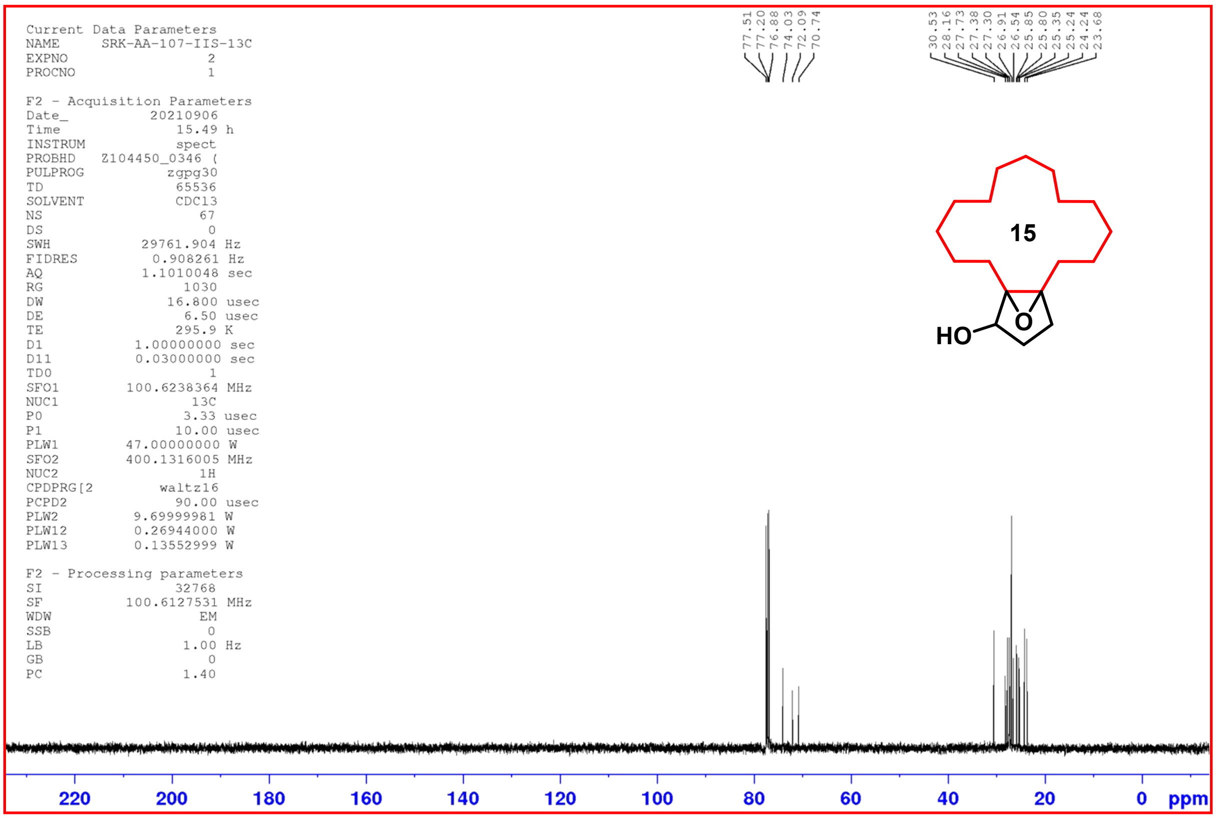

^{1}**H NMR** (400 MHz, $CDCl_3$) of compound **29**

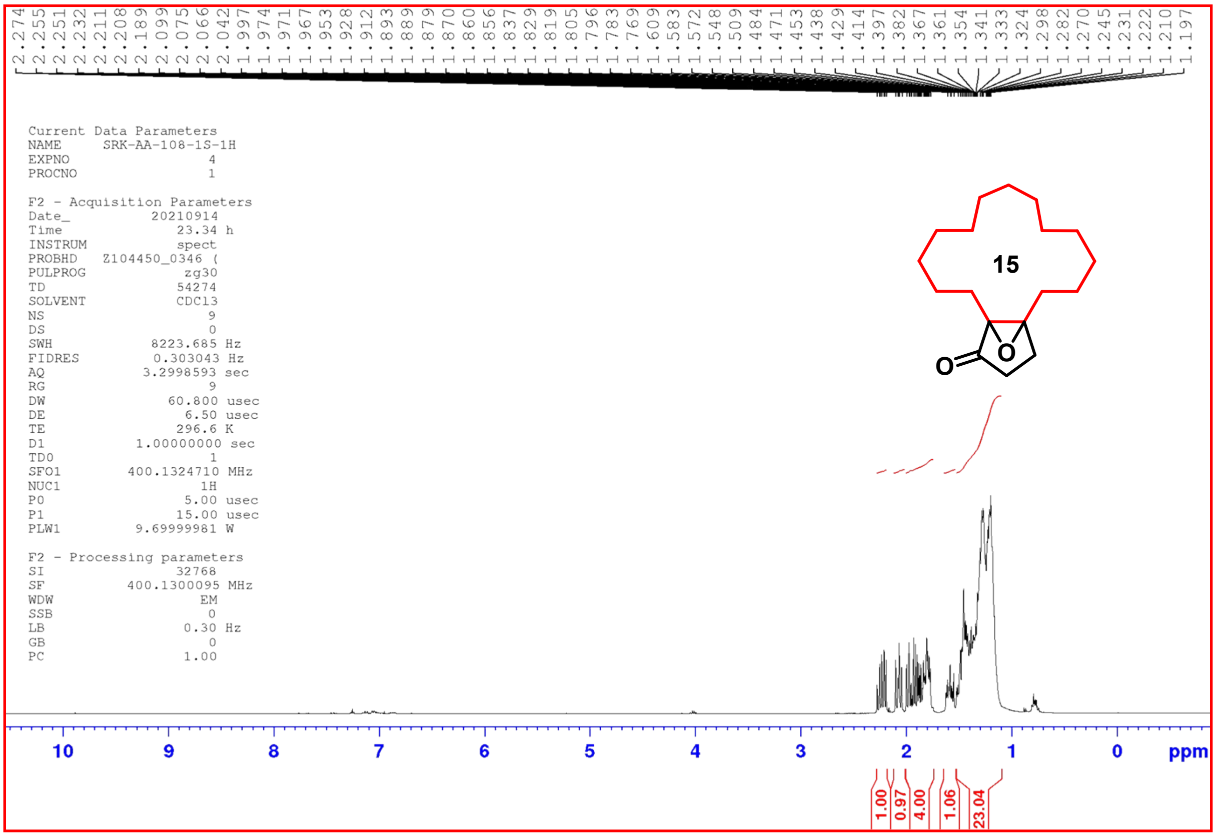

^{13}C NMR (100 MHz, $CDCl_3$) of compound **29**

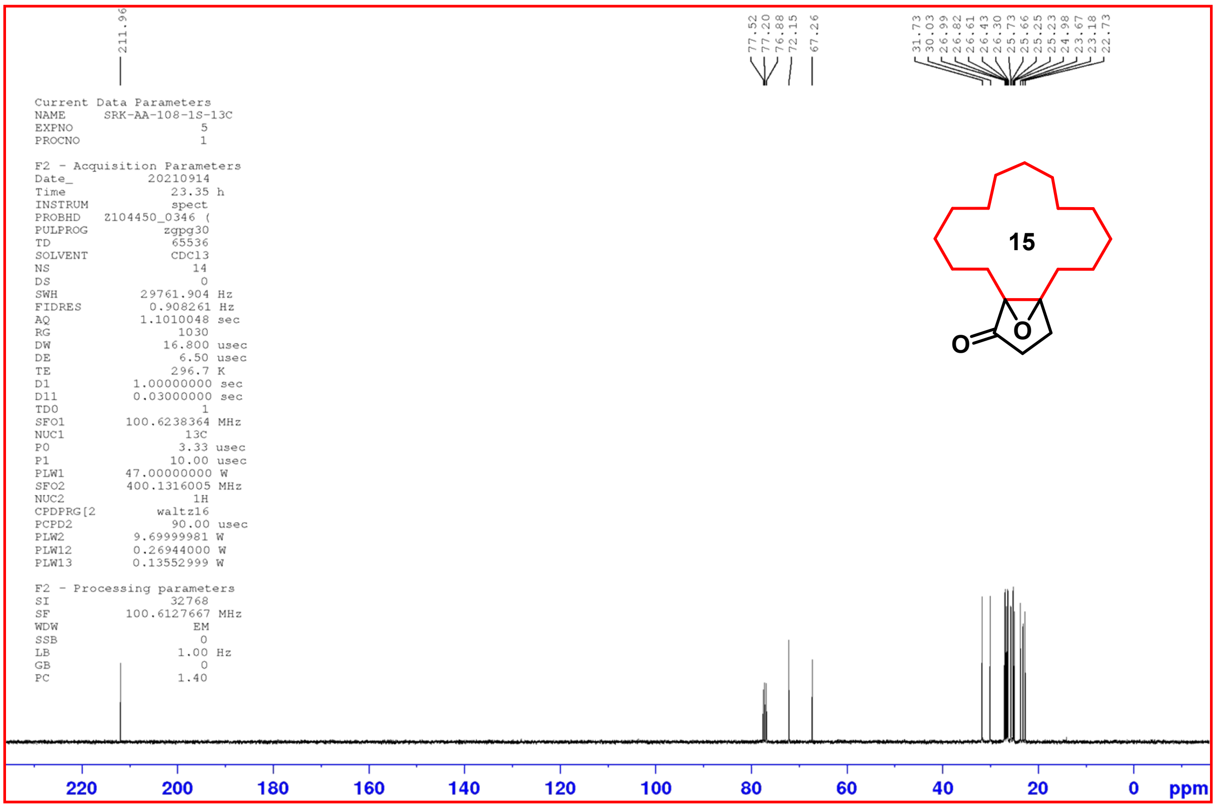

^{1}H NMR (400 MHz, $CDCl_3$) of compound **30**

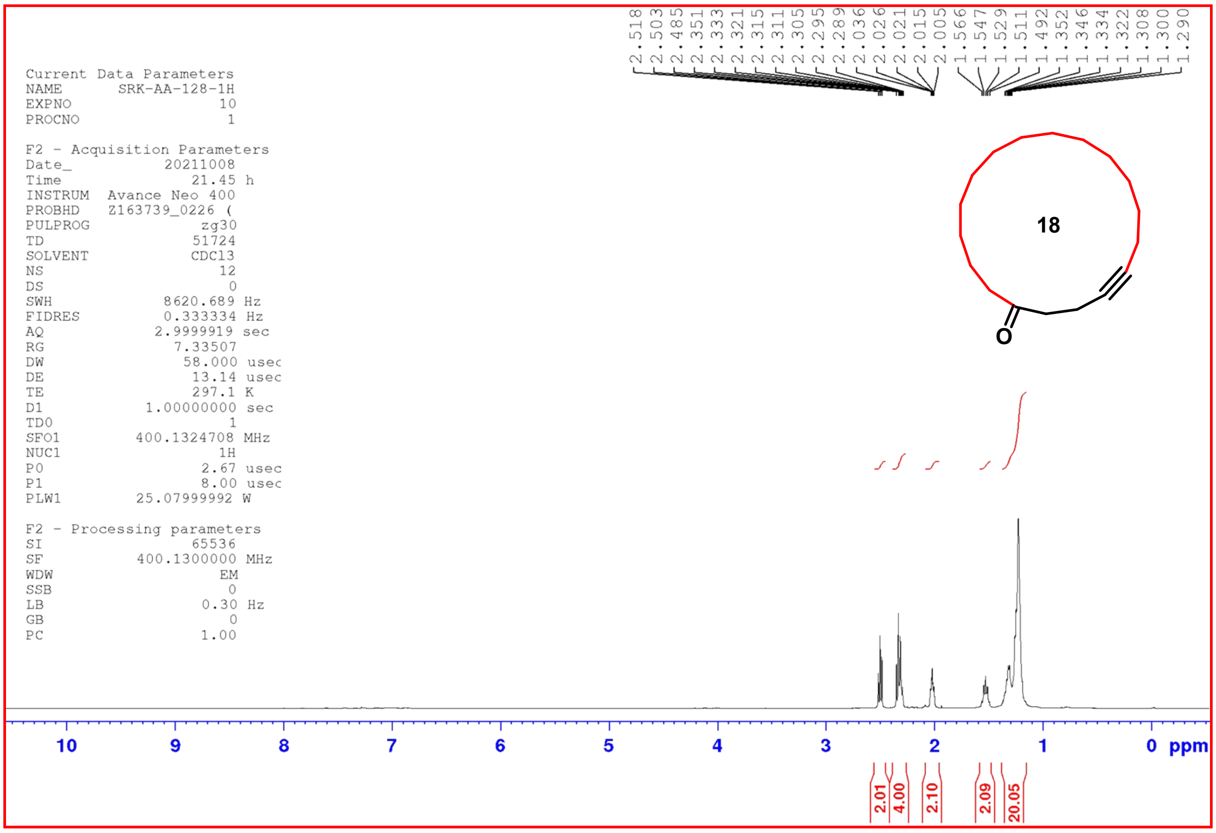

^{13}**C NMR** (100 MHz, $CDCl_3$) of compound **30**

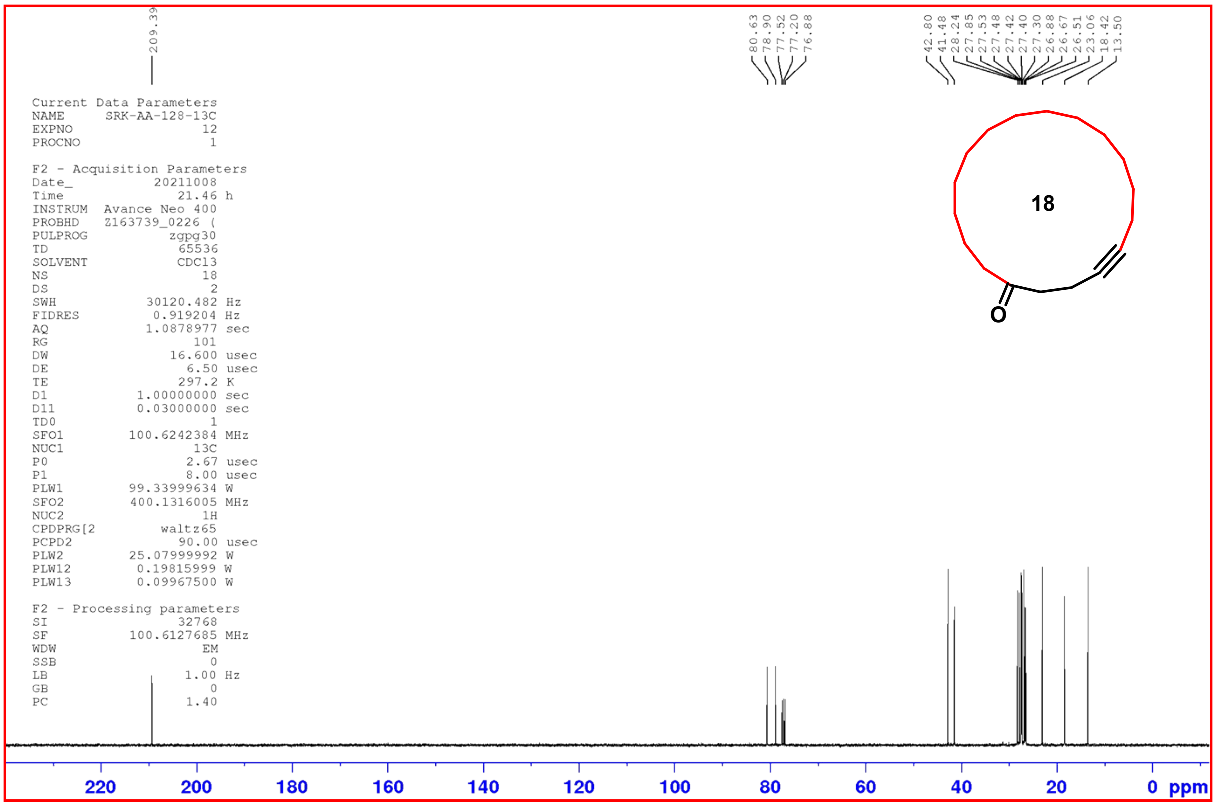

^{1}**H NMR** (500 MHz, $CDCl_3$) of compound **31**

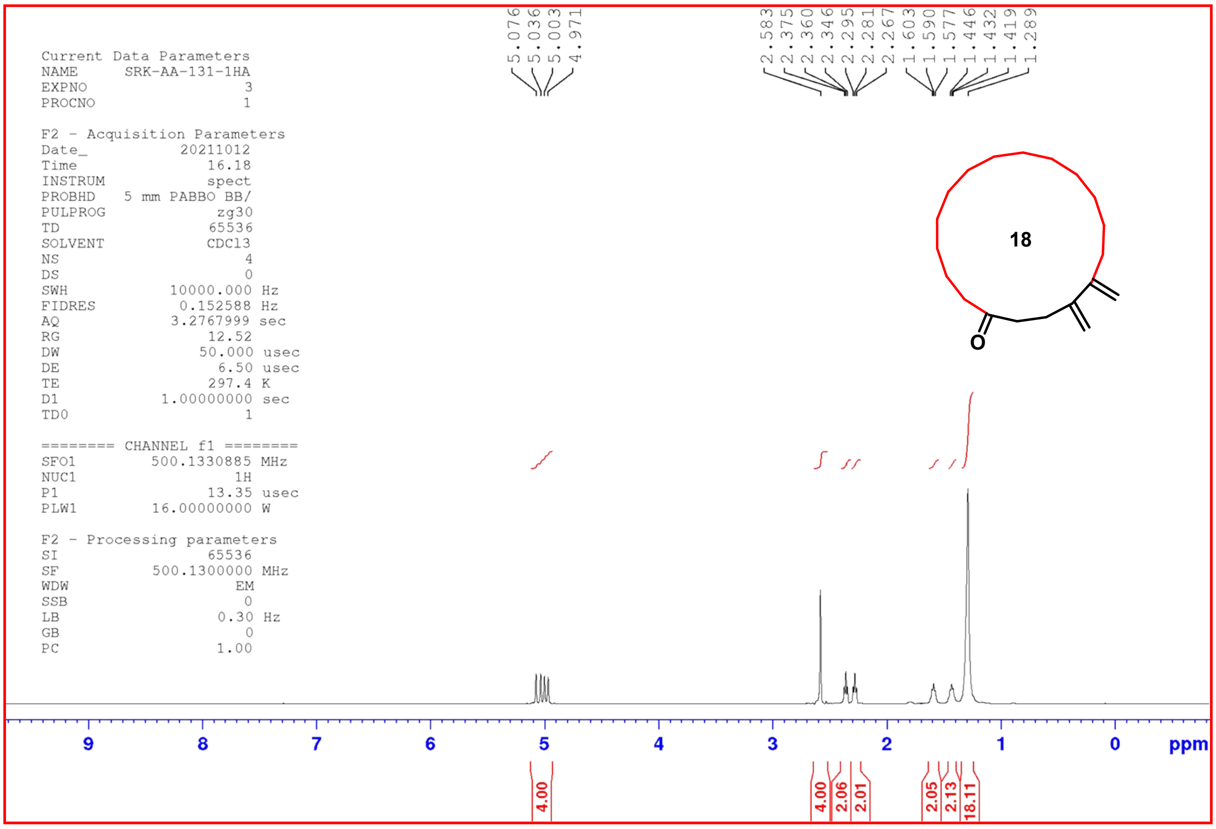

^{13}C NMR (125 MHz, $CDCl_3$) of compound **31**

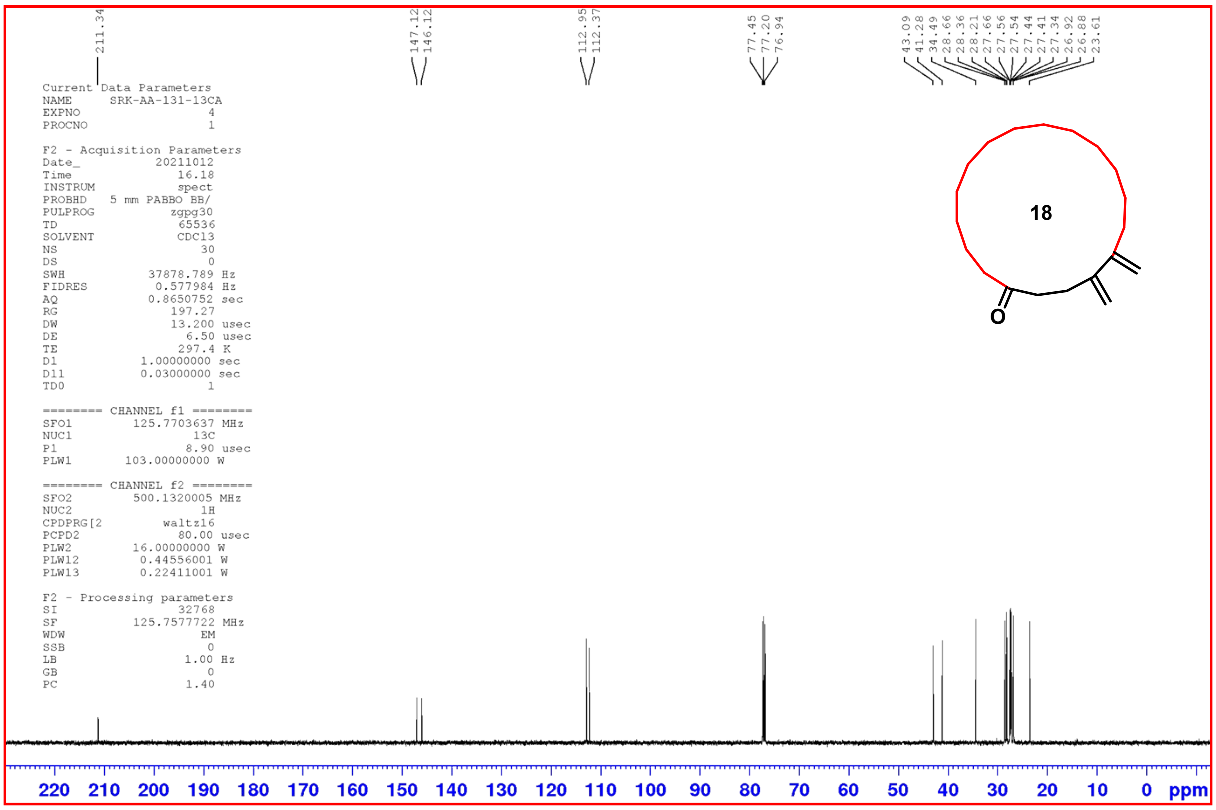

^{1}H NMR (500 MHz, $CDCl_3$) of compound **32a**

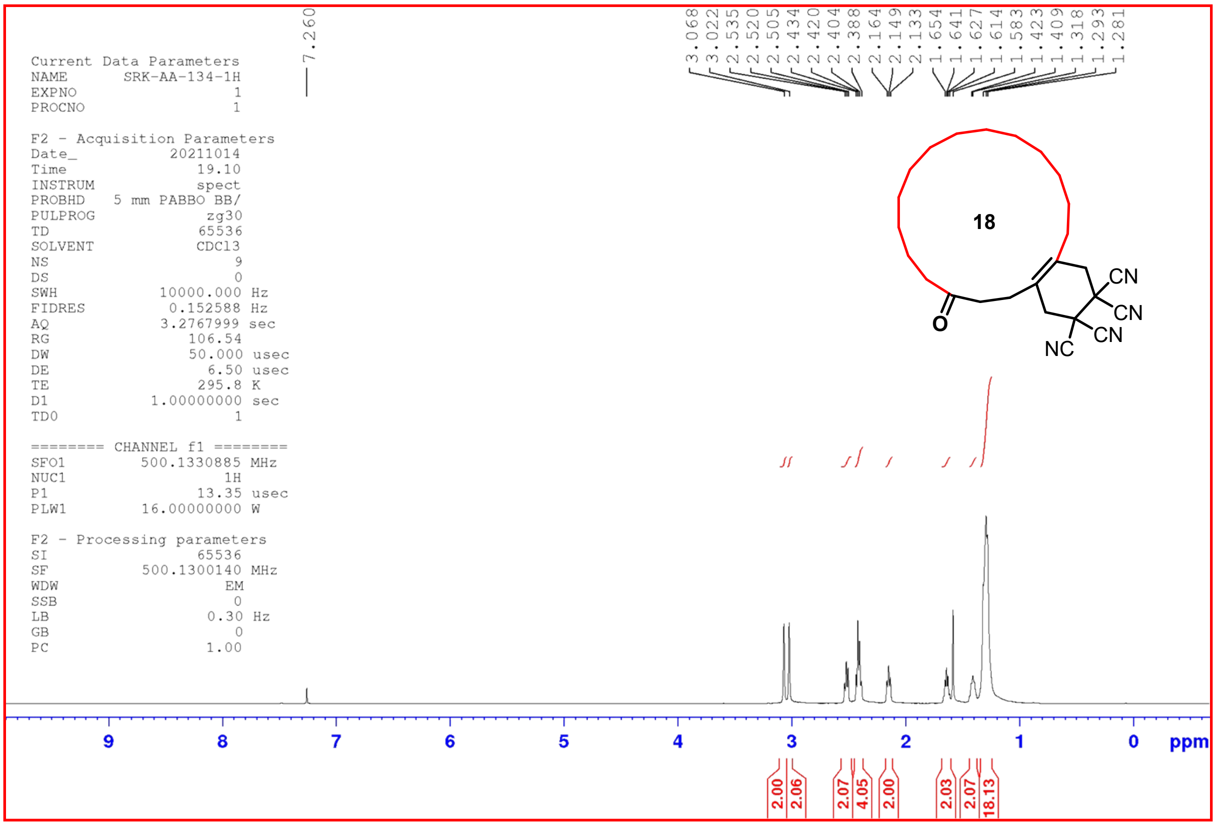

^{13}C NMR (125 MHz, $CDCl_3$) of compound **32a**

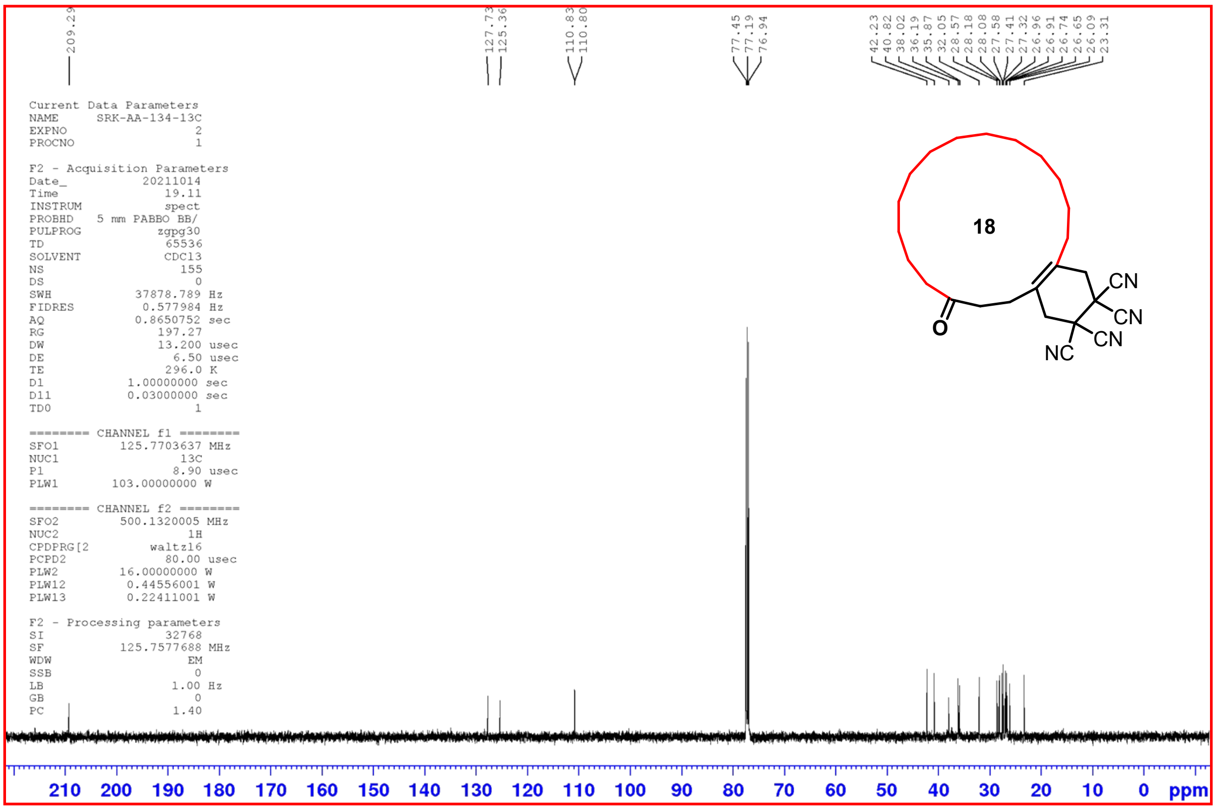

^{1}H NMR (500 MHz, $CDCl_3$) of compound **32b**

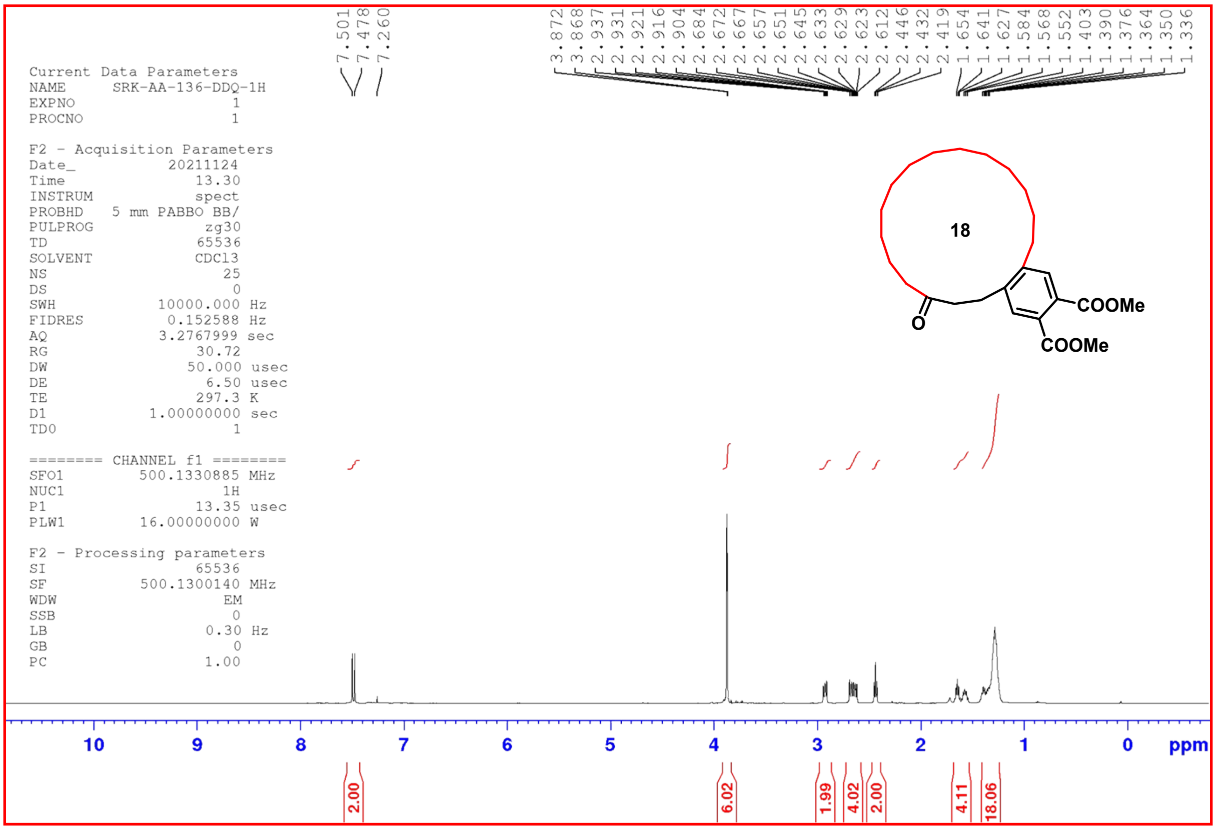

^{13}**C NMR** (125 MHz, $CDCl_3$) of compound **32b**

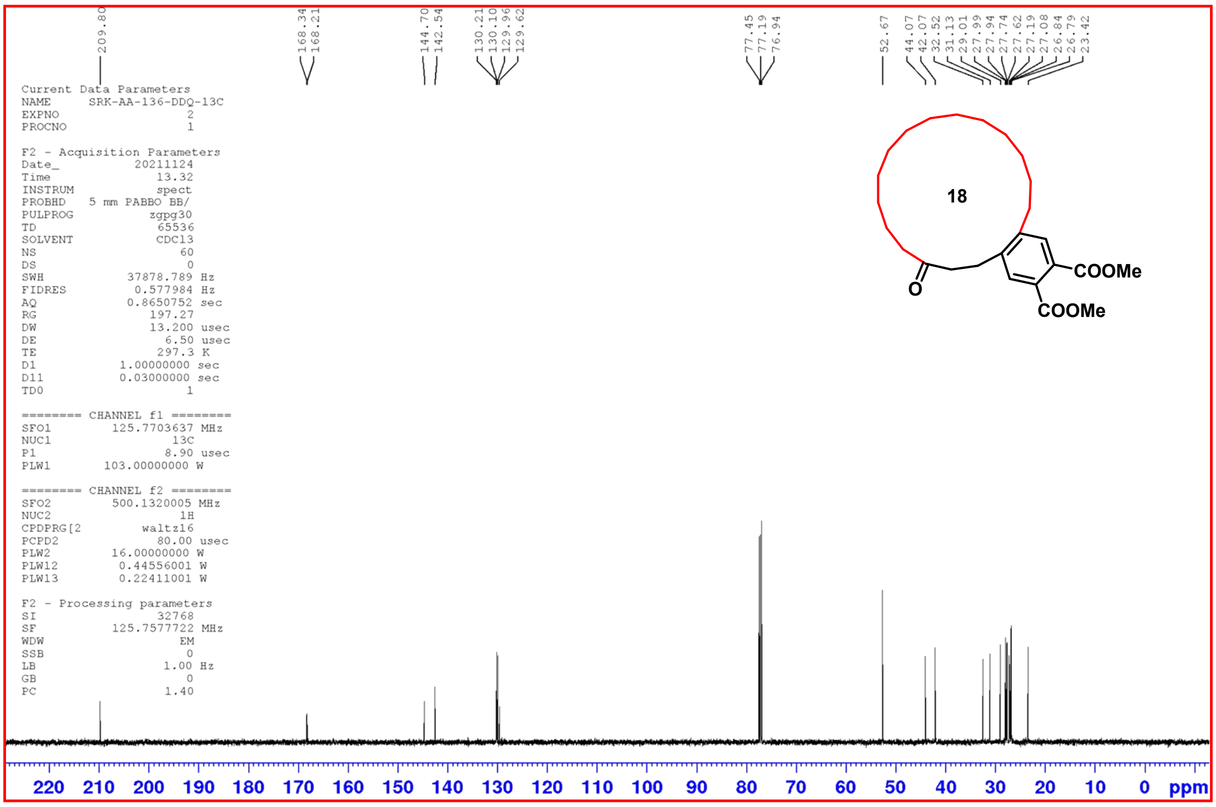

^{1}**H NMR** (400 MHz, $CDCl_3$) of compound **32c**

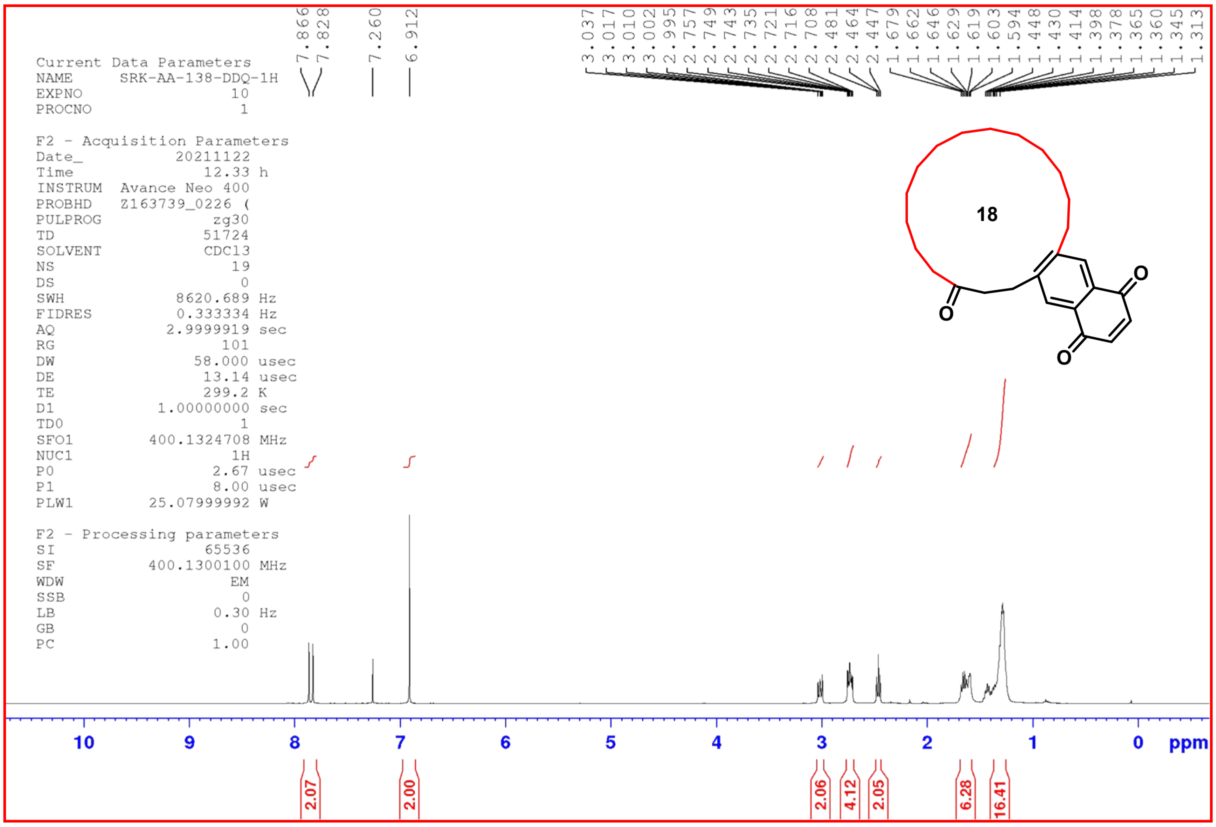

^{13}**C NMR** (100 MHz, $CDCl_3$) of compound **32c**

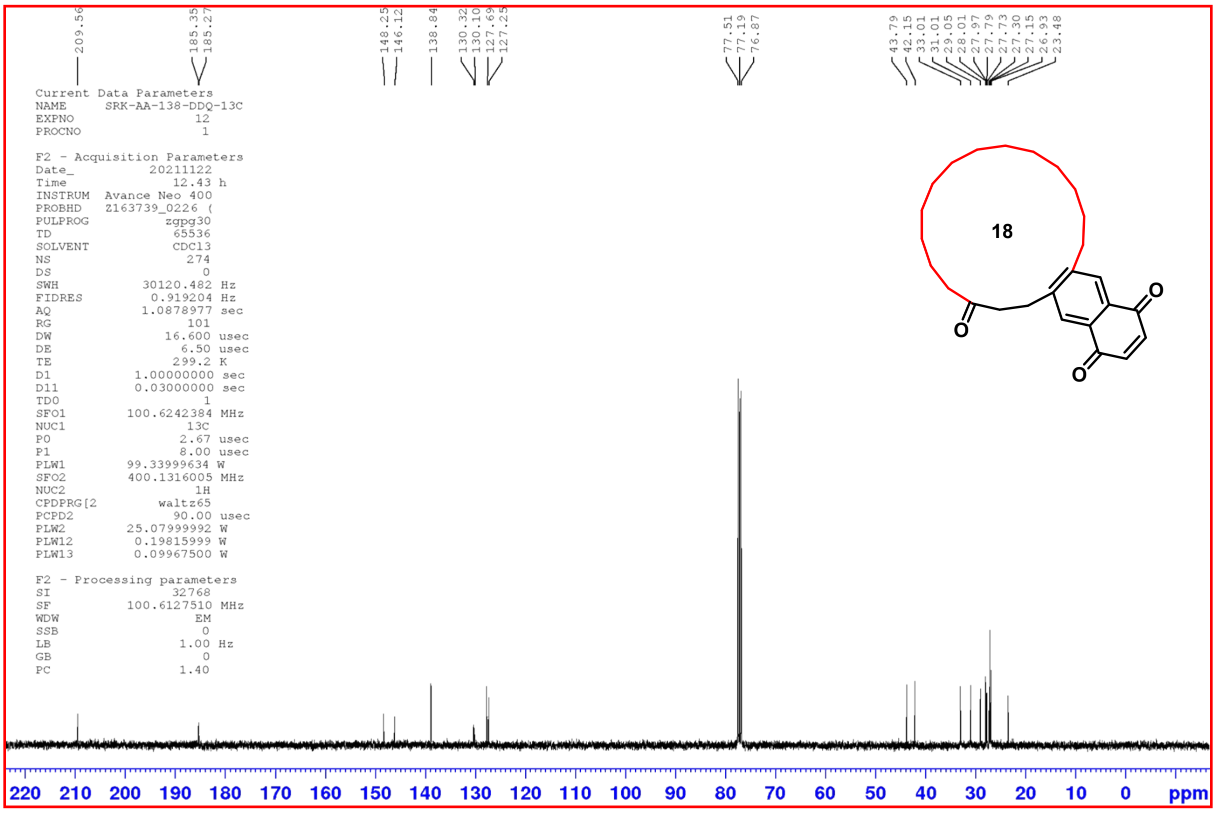

^{1}**H NMR** (500 MHz, $CDCl_3$) of compound **32d**

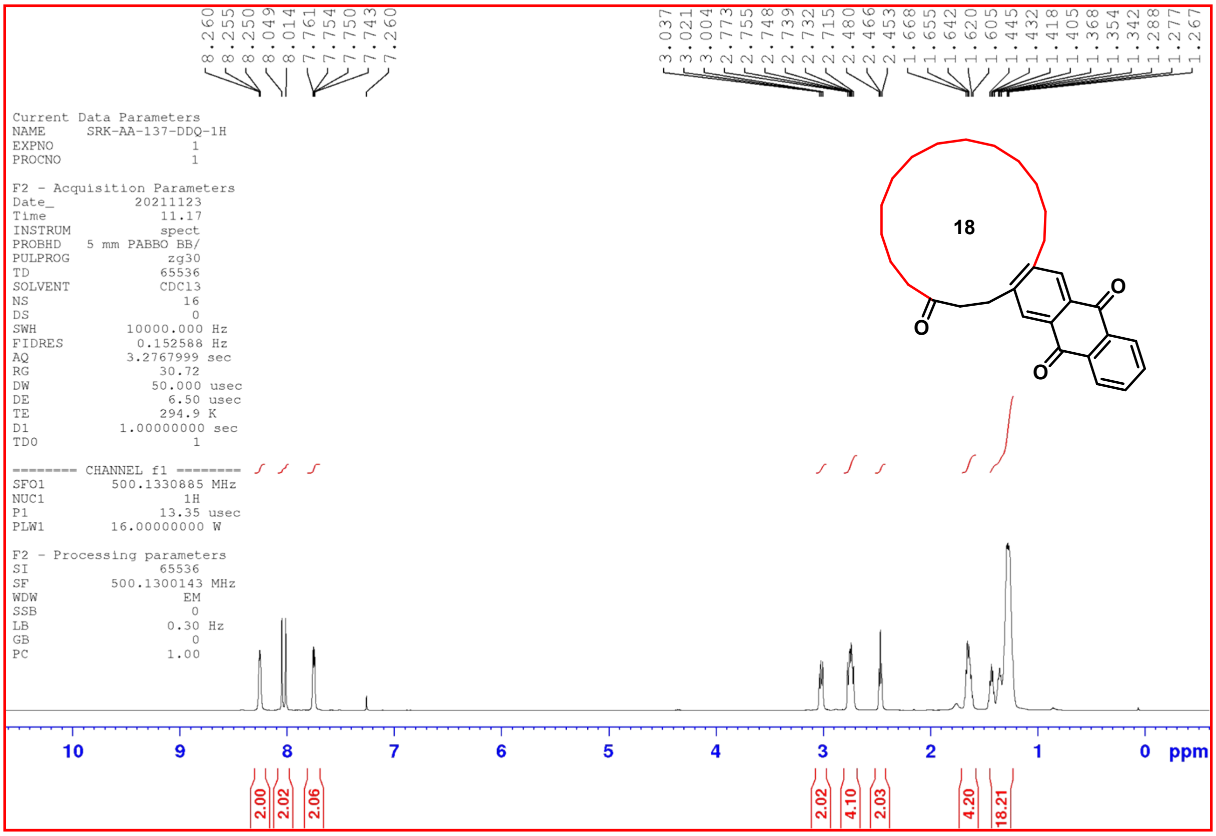

^{13}C NMR (125 MHz, $CDCl_3$) of compound **32d**

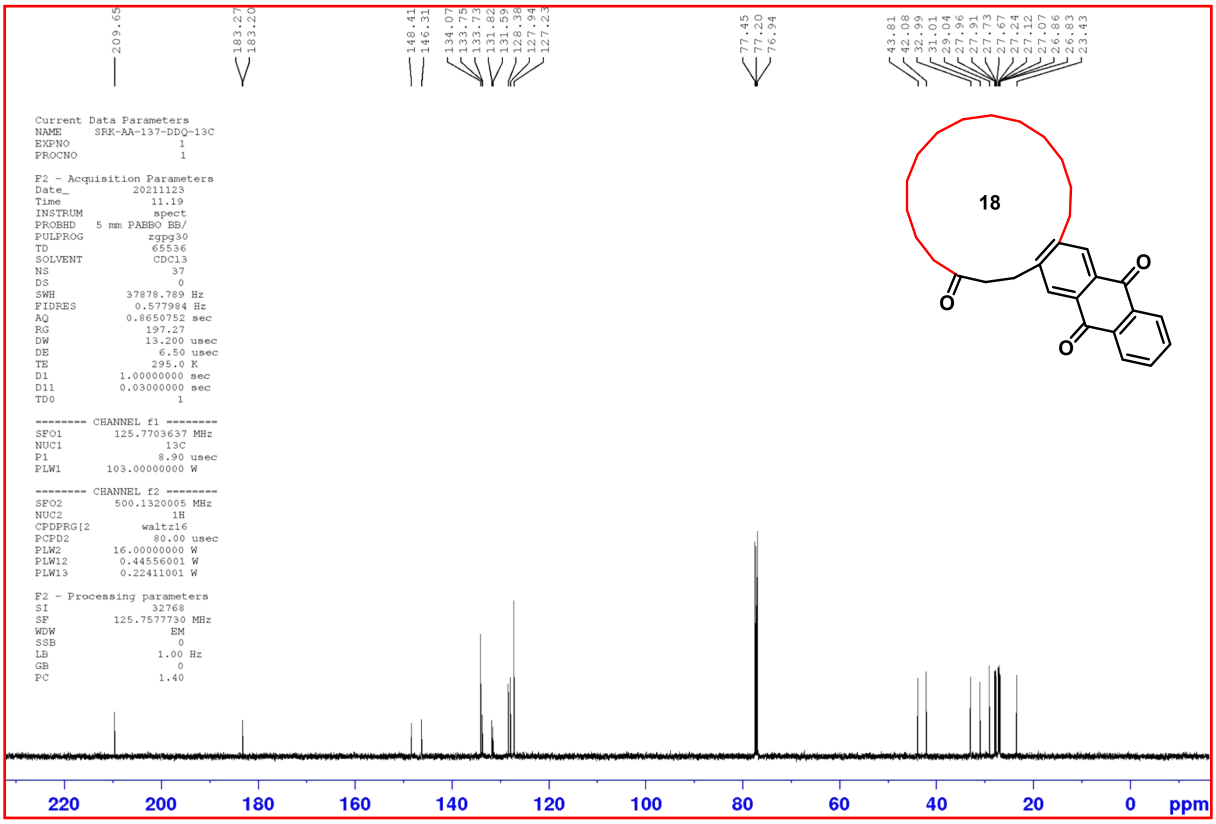

Supplement: RA-012-D2RA01458K-s001 [file RA-012-D2RA01458K-s001.pdf]
